# USP45 Represses Melanoma Development by Deubiquitinating and Stabilizing Tumor Suppressor MRGPRF

**DOI:** 10.1002/advs.202503106

**Published:** 2025-08-11

**Authors:** Wancong Zhang, Liyun Chen, Jing Zhao, Aiwei Ma, Wenqi Shi, Yiwen Zhang, Zixuan Tang, Jiarui Guo, Zaihua Xu, Jianda Zhou, Shijie Tang

**Affiliations:** ^1^ Department of Plastic Surgery and Burn Center Second Affiliated Hospital Shantou University Medical College Shantou Guangdong 515051 China; ^2^ Plastic Surgery Institute of Shantou University Medical College Shantou Guangdong 515051 China; ^3^ Shantou Plastic Surgery Clinical Research Center Shantou Guangdong 515051 China; ^4^ Research Center of Translational Medicine Second Affiliated Hospital of Shantou University Medical College Shantou Guangdong 515051 China; ^5^ College of Medicine Shantou University Shantou Guangdong 515051 China; ^6^ Department of Plastic and Reconstructive Surgery Central South University Third Xiangya Hospital Changsha Hunan 410000 China

**Keywords:** melanoma, MRGPRF, ubiquitination, USP45

## Abstract

Melanoma, a highly malignant skin cancer, has seen a rising incidence and death toll. MRGPRF is a novel melanoma suppressor that inhibits the PI3K/AKT pathway. However, the regulation of MRGPRF in melanoma remains unclear. Here, 40 ubiquitin‐specific proteases (USPs) are screened and USP45 is identified as a significant stabilizer of MRGPRF. Immunohistochemistry on melanoma patient biopsies demonstrates that USP45 expression is markedly reduced in melanoma tissues compared to adjacent noncancerous epidermis. Bioinformatic analyses corroborate that USP45 mRNA levels are downregulated in melanoma, and low USP45 expression is associated with poor patient prognosis. Functional assays demonstrate that USP45 overexpression inhibits melanoma cell malignancy, whereas USP45 knockdown promotes it. Mechanistically, USP45's catalytic domain directly binds to the N‐terminal of MRGPRF and stabilizes MRGPRF, likely by removing its K63‐linked ubiquitination in melanoma cells. The antimelanoma effects of USP45 are mitigated by MRGPRF depletion, while MRGPRF overexpression rescues the enhanced malignant phenotype induced by USP45 deficiency. In vivo, a melanoma xenograft mouse model shows that USP45 overexpression significantly impairs melanoma progression. These findings establish USP45 as a melanoma suppressor, at least partially through its stabilization of MRGPRF, highlighting a novel mechanism in melanoma pathogenesis and suggesting that USP45 agonists may serve as potential therapeutic agents.

## Graphical abstract

USP45 acts as a melanoma suppressor by stabilizing MRGPRF, which weakens the PI3K/AKT pathway, suppresses tumor growth, and reduces melanoma cell migration and invasion.

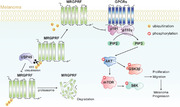

## 1 Introduction

Melanoma, which originates from cancerous melanocytes, is a malignant skin cancer.^[^ ^1^ ^]^ Although melanoma is less common than other types of skin cancer, it is more dangerous due to its higher likelihood of metastasis if not detected and treated promptly.^[^ ^2^ ^]^ Melanoma can develop anywhere on the body, but is more prone to occur in sun‐exposed areas such as the back, limbs, and face.^[^ ^3^, ^4^ ^]^ The prevalence of melanoma has continued to rise over the decades, making it a significant threat to public health.^[^ ^5^ ^]^

Melanoma occurrence also exhibits a geographic pattern, with a significantly higher rate in developed countries.^[^ ^6^ ^]^ It particularly affects the older Caucasian male population.^[^ ^5^ ^]^ Other risk factors associated with melanoma include UV exposure, family history, defective immune system, and more.^[^ ^7^ ^]^ Although novel treatments have substantially improved the prognosis for melanoma patients,^[^ ^8^, ^9^, ^10^ ^]^ insufficient drug responsiveness, the gradual development of drug resistance, and other factors may lead to treatment failure,^[^ ^11^, ^12^ ^]^ making melanoma the most lethal skin cancer globally.^[^ ^6^ ^]^ Therefore, new therapies are urgently needed to combat this fast‐growing malignancy.

The molecular mechanisms governing melanoma development have been intensively investigated, and various genetic mutations have been identified as correlating with melanoma development.^[^ ^13^, ^14^, ^15^ ^]^ For instance, mutations of BRAF, especially the V600E variant, which lead to activation of the MAPK pathway, can be detected in ≈50% of melanoma cases.^[^ ^16^ ^]^ The PI3K/AKT signaling pathway is another pathway that is frequently overactivated in melanoma, primarily due to the amplification of *AKT3* genes and loss of PTEN function.^[^ ^17^, ^18^ ^]^ However, the precise processes responsible for the activation of these crucial carcinogenic signaling pathways remain incompletely comprehended, and new regulators are continuously being uncovered. Among these, MRGPRF is a newly identified melanoma suppressor whose expression is downregulated in melanoma.^[^ ^19^ ^]^

MRGPRF is a transmembrane protein belonging to the Mas‐related G protein‐coupled receptor family.^[^ ^20^ ^]^ Originally identified in dorsal root ganglia,^[^ ^21^ ^]^ these receptors regulate the function of neurons and muscle cells.^[^ ^22^, ^23^, ^24^ ^]^ However, the precise function of MRGPRF remains poorly defined. In melanoma cells, an elevated level of MRGPRF has been shown to suppress proliferation and migration in vitro, as well as inhibit the growth and metastasis of melanoma xenografts in vivo, possibly by reducing activation of the PI3K/AKT pathway.^[^ ^19^ ^]^ Mechanistically, MRGPRF interacts with the N‐terminal domain (residues 1–216) of p110γ, directly competing with p101 for binding at this site. This competitive interaction prevents formation of the functional Class IB PI3K complex (p110γ/p101), consequently inhibiting downstream signaling activation. The low expression of MRGPRF mRNA and protein in melanoma may be attributed to hypermethylation of its promoter region, which causes decreased transcription.^[^ ^19^ ^]^ Nevertheless, the mechanisms that regulate MRGPRF protein metabolism and whether their dysregulation plays a role in the reduced MRGPRF protein levels in melanoma cells remain unclear.

Proteostasis is a complex process that cells utilize to regulate the concentration, structure, and location of proteins.^[^ ^25^ ^]^ It involves various pathways coordinating protein synthesis, folding, transport, and degradation.^[^ ^26^ ^]^ Eukaryotic cells utilize two major pathways—the ubiquitin–proteasome system and lysosomal proteolysis—to degrade proteins.^[^ ^27^ ^]^ While proteasomes primarily break down short‐lived and soluble misfolded proteins via the ubiquitin–proteasome system, lysosomes degrade long‐lived proteins and insoluble protein aggregates.^[^ ^28^ ^]^ Dysregulated proteostasis is believed to play a critical role in melanoma progression.^[^ ^29^ ^]^

Ubiquitination involves the attachment of ubiquitin to specific proteins through a series of enzymatic reactions.^[^ ^30^ ^]^ Beyond marking proteins for degradation by the proteasome complex, ubiquitination also regulates their cellular distribution and activity.^[^ ^31^ ^]^ Ubiquitin can bind to the cysteine residue of substrates at various lysine residues (K), such as K48 and K63, each serving distinct functions.^[^ ^32^ ^]^

Ubiquitination can be reversed by deubiquitinases (DUBs), which cleave the ubiquitin chain from targeted proteins. DUBs are categorized into multiple subfamilies based on their structure. Among these, ubiquitin‐specific proteases (USPs), also known as ubiquitin‐specific peptidases, form the largest DUB subfamily.^[^ ^33^ ^]^ The human genome encodes more than 50 USPs, and these enzymes remove ubiquitin chains from substrate proteins using their catalytic domain, which is functionally conserved across the USP family.^[^ ^33^, ^34^ ^]^

USPs play diverse roles in various physiological and pathological processes, including tumorigenesis,^[^ ^35^ ^]^ where their involvement in melanoma has been recognized. For instance, USP4 is known to be overexpressed in melanoma cells, and its knockdown has been shown to mitigate the migration of melanoma cells in vitro.^[^ ^36^ ^]^ USP7 is also overexpressed in melanoma and is negatively associated with the prognosis of melanoma patients.^[^ ^37^ ^]^ Pharmacological inhibition of USP7 inhibits melanoma growth and progression.^[^ ^37^ ^]^ However, the roles of other USPs in melanoma, along with the underlying mechanisms and their substrate proteins, remain elusive.

To investigate whether USPs influence the proteostasis of MRGPRF, thereby regulating melanoma tumorigenesis, we initially screened 40 USPs that may influence the stability of MRGPRF and identified USP45 as a potent stabilizer of MRGPRF. Next, we examined the expression of USP45 in melanoma tissues and adjacent noncancerous epidermis. We also assessed USP45 expression in melanoma and normal skin using published transcriptomic datasets and evaluated the relationship between USP45 expression and the prognosis of melanoma patients. Furthermore, we examined the role of USP45 in melanoma cell lines and a melanoma xenograft model. Additionally, we investigated the mechanism that governs the interaction between USP45 and MRGPRF in melanoma.

## 2 Experimental Section

### 2.1 Human Subjects

This study included 15 melanoma patients diagnosed and treated at the hospital between 2019 and 2023. All procedures were approved by the Ethics Committee of the IRB of Third Xiangya Hospital, Central South University (2022‐S066), and written consent was obtained from the patients. The noncancerous skin adjacent to melanoma tissues (<1 cm) was considered as control skin.

### 2.2 Cell Culture

HEK293T, HaCaT, A375, SK‐MEL‐28, SK‐MEL‐2, and A875 cell lines were obtained from IMMOCELL (Xiamen, China) and subjected to routine *Mycoplasma* contamination tests before subsequent experiments. All cells were cultured in specialized media purchased from IMMOCELL and maintained in a humidified incubator at 37 °C with 5% CO_2_. For specific experiments, the culture media were supplemented with 30 µm Cycloheximide (CHX) and/or 20 or 30 µm MG132.

### 2.3 Plasmid Construction

The coding sequences of individual USPs were cloned into the pcDNA3.1‐HA tag vector for transient overexpression. The 40 USPs examined in this study included USP1–8, USP10, 11, USP13–15, USP18–22, USP25, 26, USP27X, USP29, 30, USP32, 33, USP35, 36, USP38–40, USP44–46, USP48, 49, USP51–54, and USPL1. All these plasmids were purchased from Anti‐hela Biological Technology (Xiamen, China). The coding sequence of MRGPRF was inserted into the pmirGLO‐G4S‐Nanoluc vector, pcDNA3.1‐Flag vector, and pcDNA3.1‐His vector, and named MRGPRF‐Nanoluc, Flag‐MRGPRF, and His‐MRGPRF, respectively.

To deplete USP45 or MRGPRF, specific USP45‐ or MRGPRF‐targeting shRNAs were cloned into the pLVX‐CMV‐pGK‐puro lentiviral vector and named shUSP45‐1, shUSP45‐2, shUSP45‐3, shMRGPRF‐1, shMRGPRF‐2, and shMRGPRF‐3. A nonspecific shRNA‐containing lentiviral vector (shNC) was used as a control. The His‐Ub WT, His‐Ub K63, His‐Ub K48, His‐Ub K63R, and His‐Ub K48R were obtained from Wuhan Miaoling Technology (China). The accuracy of all plasmids was verified by sequencing. The production of lentivirus was carried out by Anti‐hela Biological Technology, employing the four‐plasmid system in HEK293T cells.^[^ ^38^ ^]^ Virus Multiplicity of Infection (MOI) was determined by TaqMan real‐time polymerase chain reaction (PCR). The catalog numbers for purchased plasmids are shown in Table S1 (Supporting Information). The primer sequence for plasmid construction is listed in **Table** **1**.

**Table 1:** The primer sequence for plasmid construction.

Primers	Sequence 5′–3′
MRGPRF‐Nanoluc‐F	TGGAGCCACCGAATTCATGGCTGGAAACTGCTCCTGGGAGG
MRGPRF‐Nanoluc‐R	CCCGAGCCACCGCCACCAGAGGAGGCGTTCCCCGGGGGAC
Flag‐MRGPRF‐F	TAGAGCTAGCGAATTCGCCACCATGGCTGGAAACTGCTCCTG
Flag‐MRGPRF‐R	CTTTGTAGTCGGATCCGGAGGCGTTCCCCGGGGGACACTGCATCTCCATG
HA‐USP45 (1‐170 aa)‐F	TAGAGAATTCGGATCCGCCACCATGCGGGTGAAAGATCCAAC
HA‐USP45 (1‐170 aa)‐R	CATAAGGGTAAAGCTTTTCACAAAGTTTCATGATTC
HA‐USP45 (61‐814aa)‐F	TAGAGAATTCGGATCCGCCACCATGGTTTGCTCAGAATGTTTAAAAG
HA‐USP45 (61‐814aa)‐R	CATAAGGGTAAAGCTTTAATACTCTTTCATAGAAAAG
HA‐USP45 (190‐814aa)‐F	TAGAGAATTCGGATCCGCCACCATGAGAGGAATTACAAATTTAGG
FLAG‐MRGPRF (182‐343aa)‐F	TACCGAGCTCGGATCCGCCACCATGTGCGTGTTCCTGGGCCGCGG
FLAG‐MRGPRF (182‐343aa)‐R	GCCCTCTAGACTCGAGGGAGGCGTTCCCCGGGGGAC
FLAG‐MRGPRF (1‐181aa)‐F	TACCGAGCTCGGATCCGCCACCATGGCTGGAAACTGC
FLAG‐MRGPRF (1‐181aa)‐R	GCCCTCTAGACTCGAGGAAGTAGTTGTGCAGGCAGG
shMRGPRF‐1‐F	CCGGGCTTCTCCATCAAGAGGAACCCTCGAGGGTTCCTCTTGATGGAGAAGCTTTTT
shMRGPRF‐1‐R	AATTAAAAAGCTTCTCCATCAAGAGGAACCCTCGAGGGTTCCTCTTGATGGAGAAGC
shMRGPRF‐2‐F	CCGGGGATCGACTGGTTCCTCTTCTCTCGAGAGAAGAGGAACCAGTCGATCCTTTTT
shMRGPRF‐2‐R	AATTAAAAAGGATCGACTGGTTCCTCTTCTCTCGAGAGAAGAGGAACCAGTCGATCC
shMRGPRF‐3‐F	CCGGGCAAGGCGGTGTTCTCCATCCCTCGAGGGATGGAGAACACCGCCTTGCTTTTT
shMRGPRF‐3‐R	AATTAAAAAGCAAGGCGGTGTTCTCCATCCCTCGAGGGATGGAGAACACCGCCTTGC
shUSP45‐1‐F	CCGGGGTGAAAGATCCAACTAAAGCCTCGAGGCTTTAGTTGGATCTTTCACCTTTTT
shUSP45‐1‐R	AATTAAAAAGGTGAAAGATCCAACTAAAGCCTCGAGGCTTTAGTTGGATCTTTCACC
shUSP45‐2‐F	CCGGGATGAATGAGATCAAAGAAAGCTCGAGCTTTCTTTGATCTCATTCATCTTTTT
shUSP45‐2‐R	AATTAAAAAGATGAATGAGATCAAAGAAAGCTCGAGCTTTCTTTGATCTCATTCATC
shUSP45‐3‐F	CCGGCATTCTTCATCTAAAGATAAGCTCGAGCTTATCTTTAGATGAAGAATGTTTTT
shUSP45‐3‐R	AATTAAAAACATTCTTCATCTAAAGATAAGCTCGAGCTTATCTTTAGATGAAGAATG
shNC‐F	CCGGTTCTCCGAACGTGTCACGTTTCTCGAGAAACGTGACACGTTCGGAGAATTTTT
shNC‐R	AATTAAAAATTCTCCGAACGTGTCACGTTTCTCGAGAAACGTGACACGTTCGGAGAA
USP45WT‐F	GGACTCAGATCTCGAGGCCACCATGCGGGTGAAAGATCCAAC
USP45C199A‐R1	CTGCATTAAAAAAGGCAGTATTTCCTAAATTTGTAATTC
USP45C199A‐F1	TACTGCCTTTTTTAATGCAGTCATGCAG
USP45C199A‐R2	AATCTGGTACGTCGTATGGGTATAATACTCTTTCATAGAAAAG
USP45WT‐R	TAGAGTCGCGGGATCCTTAAGCGTAATCTGGTACGTCGTATGGG

### 2.4 Cell Transfection and Virus Infection

Nonviral plasmid was transfected using Lipofectamine 2000 (Invitrogen, USA) or ExFect Transfection Reagent (T101‐01, Vazyme, China). For lentivirus infection, melanoma cells at 80% confluence were cultured with diluted virus at an optimized MOI of 10 in the presence of 10 µg mL^−1^ polybrene. Subsequently, the virus‐containing medium was replenished once after 24 h of culture. For stable cell screening, puromycin was added to the medium at a final concentration of 2 µg mL^−1^ after 72 h of initial infection, and the medium was refreshed every other day. After 7 days of screening, the surviving cells were cultured in puromycin‐free medium and collected for subsequent analyses.

### 2.5 Bioinformatic Analysis

The TCGA‐SKCM RNA‐seq dataset including 1 control and 472 skin melanoma samples was accessed from https://portal.gdc.cancer.gov/projects/TCGA‐SKCM. The relevant clinic features including tumor stages and survival status were downloaded from the Xena database (https://xena.ucsc.edu/). Additionally, transcriptomic data of 604 skin_not_sun_exposed_suprapubic and 701 skin_sun_exposed_lower_leg samples were obtained from the GTEx database (https://www.gtexportal.org/). For the comparison of USP45 expression between melanoma and control skin samples, unpaired *t*‐test was utilized. To evaluate the relationship between USP45 expression and patient survival, the R packages “survminer” (v0.4.9) and “survival” (v3.5‐7) were employed to calculate the Optimal Cutpoint and generate the Kaplan–Meier survival curves, respectively. Samples with 0 or unknown survival time were excluded from the survival analysis.

### 2.6 7‐Aminoactinomycin D (7‐AAD) Cell Cycle Analysis

To assess the cell cycle, 7‐AAD assays were performed using a kit from Beyotime (C1053S, China). Briefly, ≈1 × 10^6^ melanoma cells were mixed with 1 mL working solution prepared from the kit, and the resulting single‐cell suspension samples were incubated at 37 °C for 10 min. Subsequently, the samples were subjected to flow cytometry analysis using a NovoCyte FACS cytometer and its accompanying NovoExpress Software (Agilent, USA).

### 2.7 Annexin V–Fluorescein Isothiocyanate (FITC)/PI analysis

To assess cell apoptosis, the Annexin V–FITC/PI apoptosis detection kit (C1062S, Beyotime) was utilized according to the manufacturer's instructions. Briefly, 1 × 10^5^ cells were resuspended in 195 µL Annexin V–FITC binding solution, followed by gentle mixing with 5 µL Annexin V–FITC solution and 10 µL PI solution sequentially. The resulting cell suspensions were incubated in the dark at room temperature (RT) for 15 min and subjected to cytometry analysis.

### 2.8 MTT Assay

In compliance with the manufacturer's instructions, the MTT test was carried out to assess cell viability using a detection kit. Melanoma cells were seeded at 10 000 cells per well into 96‐well plates and cultured for the indicated time. Next, 10 µL of MTT solution (5 mg mL^−1^) was put into the cell culture, and the cells were left to incubate for 4 h at 37 °C. 150 µL of dimethyl sulfoxide was then added to each well, and the plates were shaken on a shaker for 10 min. Finally, the plate was placed on a microplate reader to read the OD value of each well at 490 nm.

### 2.9 Colony Formation Assay

Each well of 6‐well plates was seeded with 1000 cells, which were then cultivated for 14 days. Subsequently, the cells were fixed in 70% ethanol for 10 min and incubated with 1 mL of 1× Giemsa staining solution (C0133, Beyotime) for 5 min at RT. After removing the staining solution, the cells underwent two phosphate‐buffered saline (PBS) washes. The stained cell colonies were photographed and quantified using ImageJ software (NIH, USA).

### 2.10 Transwell Assay

A total of 6 × 10^4^ cells were seeded into the upper compartment of the transwell chamber (Corning, USA), while the lower chamber contained 700 µL of full medium. After a 48 h period of incubation, the cells were exposed to methanol at 25 °C for 30 min. Afterward, the cells were subjected to staining using a 0.1% crystal violet solution for 10 min. Ultimately, an inverted microscope (IX73, OLYMPUS, Japan) was utilized to observe and capture images of the outcomes. The methodology for the invasion assay was the same as the migration assay, with the exception that the upper chambers were coated with 100 µL of a 1:8 diluted Matrigel (BD Sciences, USA) prior to cell seeding.

### 2.11 qPCR

To perform qPCR experiments, total cellular RNA was extracted first using the Trizol reagent (Invitrogen, USA) in compliance with the manufacturer's protocol. Then, the PrimeScript RT Master Mix (Takara, Japan) was used to synthesize cDNA. qPCR experiments were conducted with a CFX96 machine (BioRad, USA) using ChamQ SYBR qPCR Master Mix (Vazyme). The gene expression was calculated using the 2^−ΔΔCt^ algorithm, and the results were normalized using 18S rRNA. The qPCR primers are listed in **Table** **2**.

**Table 2:** qPCR primers.

Primer	Sequence 5′–3′
USP45‐qPCR‐F	TATGGTCGCCGCAGTTTCTC
USP45‐qPCR‐R	AGGGATCAGGAAGCCGAGT
MRGPRF‐qPCR‐F	GGCTTCTCCATCAAGAGGAACC
MRGPRF‐qPCR‐R	TCAGGATGGAGAACACCGCCTT
KI67‐qPCR‐F	CAAAAGACAGTGTTGCTCAGGG
KI67‐qPCR‐R	TTTCTGCCATTACGTCCAGCA
18S‐F	CGACGACCCATTCGAACGTCT
18S‐R	CTCTCCGGAATCGAA CCCTGA

### 2.12 Immunoprecipitation (IP) and Western Blot

Cellular proteins were extracted, and the proteins were quantified using a BCA kit (PA115, TIANGEN, China). For IP experiments, Dynabeads (Invitrogen) were suspended in the Antibody binding/Washing buffer provided by the IP kit (Invitrogen) and incubated with 2 µg of anti‐Flag‐tag (Proteintech, 66008‐4‐Ig) or anti‐HA‐tag (Proteintech, 66006‐2‐Ig) antibody for 15 min, followed by washing with PBS. Afterward, 20 µL of antibody‐conjugated beads that were attached to antibodies were combined with 500 µL of protein samples. The resulting mixtures were then left to incubate at a temperature of 4 °C overnight while being gently shaken. Afterward, the beads were washed with lysis buffer. Finally, the beads were pelleted, and the supernatants were collected for Western blot analysis.

Protein samples were loaded onto sodium dodecyl sulfate–polyacrylamide gel electrophoresis gels for separation. The proteins were deposited onto PVDF membranes and subsequently blocked with a 5% nonfat solution for 30 min at RT. Subsequently, the membranes were incubated with primary antibody solutions overnight at 4 °C, followed by three washes with TBST solution and incubation with secondary antibody solutions for 2 h at RT. To visualize the signal, the membrane was finally incubated with an ECL working solution (Beyotime, P0018FM), which was recorded using a ChemiDoc device (BioRad, ChemiDoc, USA). Image J (NIH, USA) was utilized to quantify the band intensity. **Table** **3** lists the primary antibodies that were employed.

**Table 3:** Antibody used for Western blot.

Target	Species	Manufacturer	Catalog	Dilution
USP45	Rabbit	Affinity Biosciences (USA)	DF4595	1:1000
MRGPRF	Rabbit	Abcam (UK)	ab254756	1:1000
MYC	Rabbit	Proteintech (China)	10828‐1‐AP	1:1000
Snail	Rabbit	Abcam	ab216347	1:1000
PI3K	Rabbit	Proteintech	20584‐1‐AP	1:1000
P‐PI3K	Rabbit	Cell Signaling Technology (USA)	#4228	1:1000
AKT	Rabbit	Proteintech	10176‐2‐AP	1:2000
P‐AKT			28731‐1‐AP	1:1000
His‐tag			10001‐0‐AP	1:1000
Flag‐tag			20543‐1‐AP	1:20 000
HA‐tag			51064‐2‐AP	1:5000
GAPDH			10494‐1‐AP	1:5000
Actin			20536‐1‐AP	1:1000
HRP‐conjugated anti‐Rabbit IgG	Goat		SA00001‐2	1:2000

### 2.13 GST Pull‐Down Assay

His‐MRGPRF, GST‐USP45, and GST proteins were purchased from Anti‐hela Biological Technology. 20 mg of His‐MRGPRF protein was thoroughly mixed with 20 mg of GST‐USP45 or GST protein, and an aliquot was denatured and centrifuged for input. The remaining mixture was incubated with 20 µL of Glutathione Sepharose 4B beads for 6 h at 4 °C with gentle inversion. Subsequently, the beads were pelleted by centrifugation. After five washes with ice‐cold PBS, the beads were incubated with 80 µL of elution buffer at 100 °C for 10 min, and the resulting mixture was centrifuged. Finally, the collected supernatant proteins were separated and detected by Western blot.

### 2.14 Dual‐Luciferase Report Assay

To determine the level of luciferase activity, the Dual‐Glo Luciferase Assay System (Promega, USA) was applied. Briefly, HEK293T cells were transfected with individual USP overexpression plasmids. After 48 h, the cells were rinsed twice and incubated with lysis buffer included in the kit for 5 min at RT. After collecting and centrifuging the resultant lysates, 20 µL of the supernatant was combined with 100 µL of the Firefly substrate solution. In the final step, a volume of 100 µL of *Renilla* substrate solution was introduced into the mixture. The activity of *Renilla* luciferase was then recorded using the Luminometer (Promega).

### 2.15 SmBiT–LgBiT Protein–Protein Interaction (PPI) Luciferase Assay

Melanoma cells were seeded into 96‐well plates at a density of 3 × 10⁴ cells per well and transfected with USP45‐SmbiT and/or MRGPRF‐LgBiT plasmids (Wuhan Miaoling Technology) using ExFect Transfection Reagent, following the manufacturer's instructions. After transfection, the cells were cultured with NanoLuc substrates (IMC‐908, IMMOCELL) for 24 h. Subsequently, the cells were lysed, and luciferase activity was measured using the Nano&Firefly‐Glo kit (MA0522, Meilunbio, China) according to the user's guide. Luciferase activity was quantified with the Microplate Luminometer Orion II (Berthold Technologies, Germany), using NanoLuc activity as the internal control.

### 2.16 Xenograft Mouse Model

Twelve nude mice (female, 6 weeks old, ≈20 g body weight) were randomly divided into two groups, each consisting of six mice. All procedures were approved by the Experimental Animal Welfare Ethics Committee (XMSB‐2022‐0022), Central South University. The mice were anesthetized with isoflurane and subcutaneously injected with either 2 × 10^6^ control or USP45‐overexpressing stable A375 cells. The size of the tumor was assessed at the given time periods, and after nine weeks of housing, the mice were euthanized, and their tumors were dissected. Half of the tumors were treated with a 4% solution of paraformaldehyde and prepared for paraffin sectioning for further analysis. The remaining halves of the tumors were lysed for protein extraction.

### 2.17 Immunohistochemistry (IHC) and TUNEL

Paraffin sections were dewaxed, rehydrated, and rinsed with PBST. Subsequently, incubation with 5% goat serum for 30 min at RT. The sections were then incubated with Ki‐67 antibody (1:2000, catalog#: 27309‐1‐AP, Proteintech) or USP45 antibody (1:100, catalog#: DF4595, Affinity Biosciences) at 4 °C overnight. Afterward, the sections were cleansed using PBST and then exposed to HRP‐conjugated goat anti‐rabbit IgG (1:2000, catalog#: SA00001‐2, Proteintech) for a duration of 40 min at RT. Finally, the sections were stained with diaminobenzidine chromogenic solution, and the nuclei were further restained with hematoxylin solution before mounting. Finally, the sections were imaged and observed with a microscope (Zeiss, Germany). The IHC results were quantified using Image‐Pro Plus (v6.0). The DeadEnd Colorimetric TUNEL System (Promega) was used to perform the TUNEL assay in accordance with the manufacturer's instructions.

### 2.18 Statistical Analysis

Data analysis and graphic presentation were performed using Image‐Pro Plus (v6.0) and GraphPad Prism (v8). Data were presented as mean ± standard deviation. The methods for statistical significance evaluation were specified in the corresponding figure legend. *p* value < 0.05 was considered statistically significant. Each data point in the bar‐dot plots represented the value of a biological replicate or the average value of three technical replicates. The MTT, dual‐luciferase, and qPCR assays were conducted with both technical and biological triplicates. The remaining assays were performed at least 3 times independently.

## 3 Results

### 3.1 USP45 Is a Potent Stabilizer of MRGPRF in HEK293T Cells

**Figure 1:**
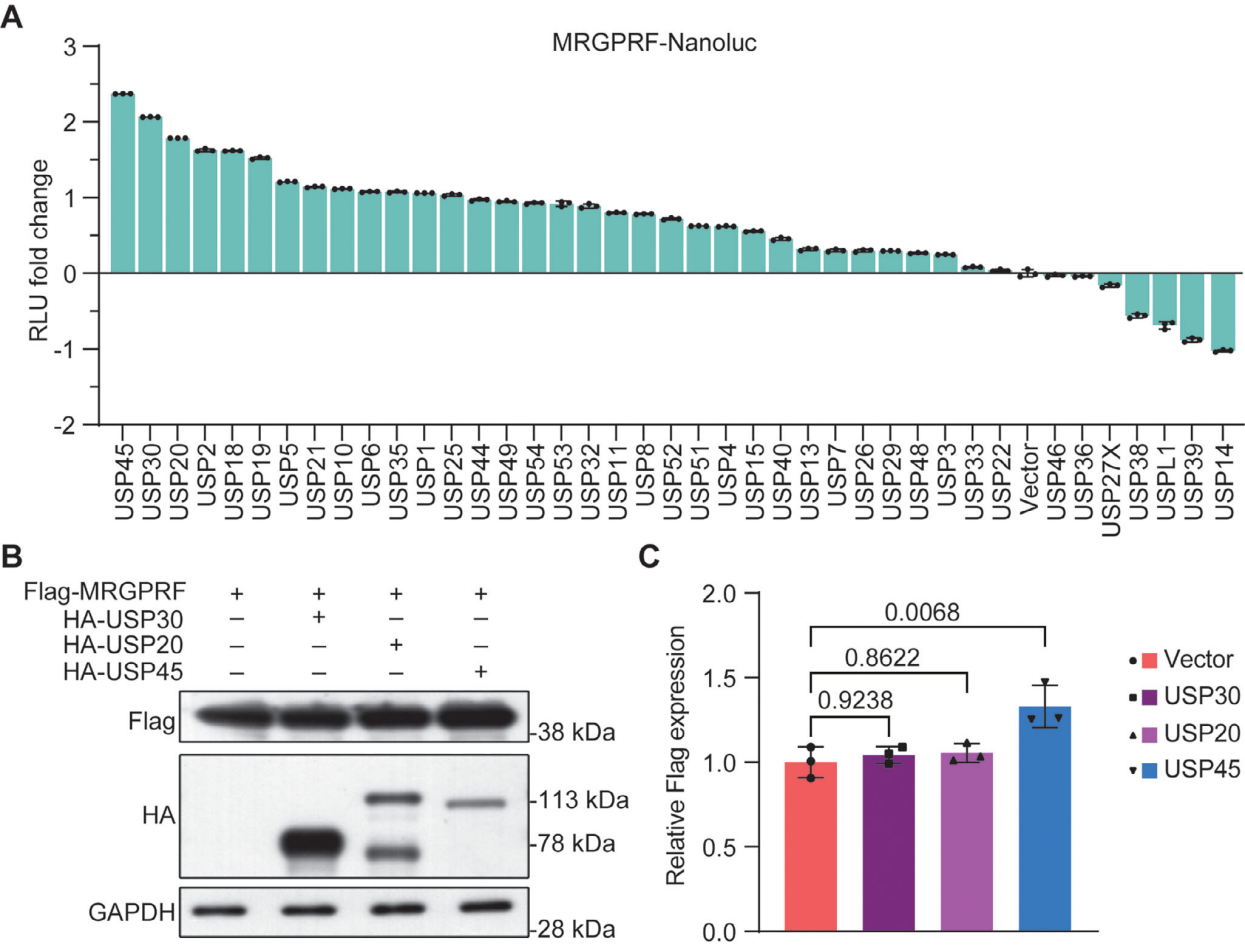
USP45 stabilizes MRGPRF in HEK293T cells. A) Luciferase assay results indicating the impact of individual USP overexpression on the stability of MRGPRF‐Nanoluc in HEK293T cells. Positive values represent increased MRGPRF‐Nanoluc activity while negative values represent reduced MRGPRF‐Nanoluc activity. B) Western blot data showing the effect of excessive USP30, USP20, or USP45 on the level of MRGPRF in HEK293T cells. C) Statistical bar graph of Western blot data shown in (B). One‐way ANOVA followed by Tukey's HSD was utilized for *p*‐value calculation.

To screen for USPs that may stabilize MRGPRF, we conjugated MRGPRF with luciferase (MRGPRF‐Nanoluc) and coexpressed it with 40 individual USPs in HEK293T cells. By measuring luciferase activity, we identified multiple USPs that enhance the activity of MRGPRF‐Nanoluc, indicating their potential ability to stabilize MRGPRF (**Figure** **1**A). Among these, USP45, USP30, and USP20 emerged as the top three stabilizers of MRGPRF‐Nanoluc. Subsequently, we cotransfected MRGPRF with USP45, USP30, or USP20. Western blot analysis revealed that only USP45 significantly increased MRGPRF levels (Figure 1B,C). These findings indicate that USP45 is a potent MRGPRF stabilizer and may play a role in melanoma.

### 3.2 Low USP45 Expression in Melanoma Tissue Predicts Poor Survival of Melanoma Patients

**Figure 2:**
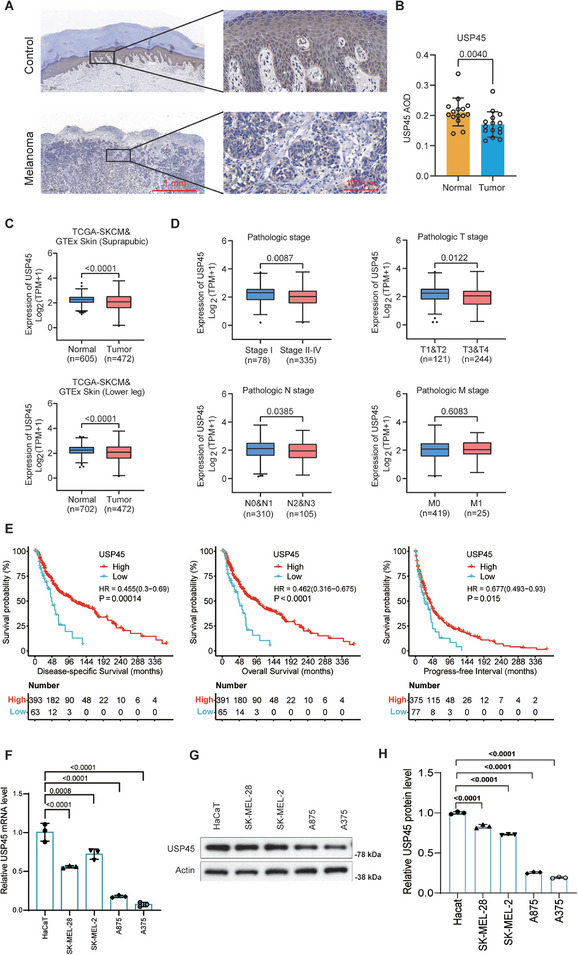
USP45 expression is reduced in melanoma and negatively associated with the prognosis of melanoma patients. A) IHC data showing the USP45 protein levels in melanoma and adjacent noncancerous epidermis. B) Statistical analysis of IHC data in (A). Fifteen samples were analyzed. *p* value is determined by unpaired bilateral Student's *t*‐test. C) Box plots illustrating the differential expression of USP45 mRNA between normal skin and melanoma samples of the indicated datasets. D) Box plots depicting USP45 expression among melanoma patients with the indicated clinicopathological features. E) Kaplan–Meier survival curves showing the prognosis of melanoma patients with high and low levels of USP45 in melanoma tissues. F,G) qPCR (F) and Western blot (G) outcomes illustrating the expression of USP45 in HaCaT keratinocytes and various melanoma cell lines. H) Statistical bar graph of Western blot data in (G). One‐way ANOVA followed by Tukey's HSD was employed for *p*‐value calculation.

To explore the role of USP45 in melanoma, we initially conducted IHC to compare USP45 protein levels between control skin and melanoma samples. The results indicated that USP45 protein levels are lower in melanoma tissues compared to the epidermis of normal skin (**Figure** **2**A,B). Consistently, USP45 expression in melanoma tissues showed a positive correlation with MRGPRF expression, both of which were reduced in these tissues (Figure 2A,B, and Figure S1A–C (Supporting Information)). Supporting these findings, our bioinformatic analysis using published transcriptomic datasets confirms that USP45 expression is significantly lower in melanoma tissues (Figure 2C). Additionally, USP45 expression in stage II–IV melanoma is lower than in stage I melanoma. USP45 levels are negatively associated with higher T and N stages, although uncorrelated with M stages of melanoma (Figure 2D). Survival analysis consistently demonstrated that melanoma patients with elevated USP45 expression exhibited a more favorable prognosis (Figure 2E). We also examined USP45 mRNA and protein levels in the immortalized human keratinocyte cell line HaCaT and human melanoma cell lines A875, SK‐MEL‐28, A375, and SK‐MEL‐2. qPCR and Western blot data findings clearly indicate that USP45 expression is reduced in these melanoma cell lines compared to HaCaT cells (Figure 2F–H). These findings indicate that USP45 expression is downregulated in melanoma and suggest that USP45 could function as a melanoma repressor.

### 3.3 USP45 Represses the Malignant Behaviors of Melanoma Cells and Attenuates the PI3K/AKT Pathway

**Figure 3:**
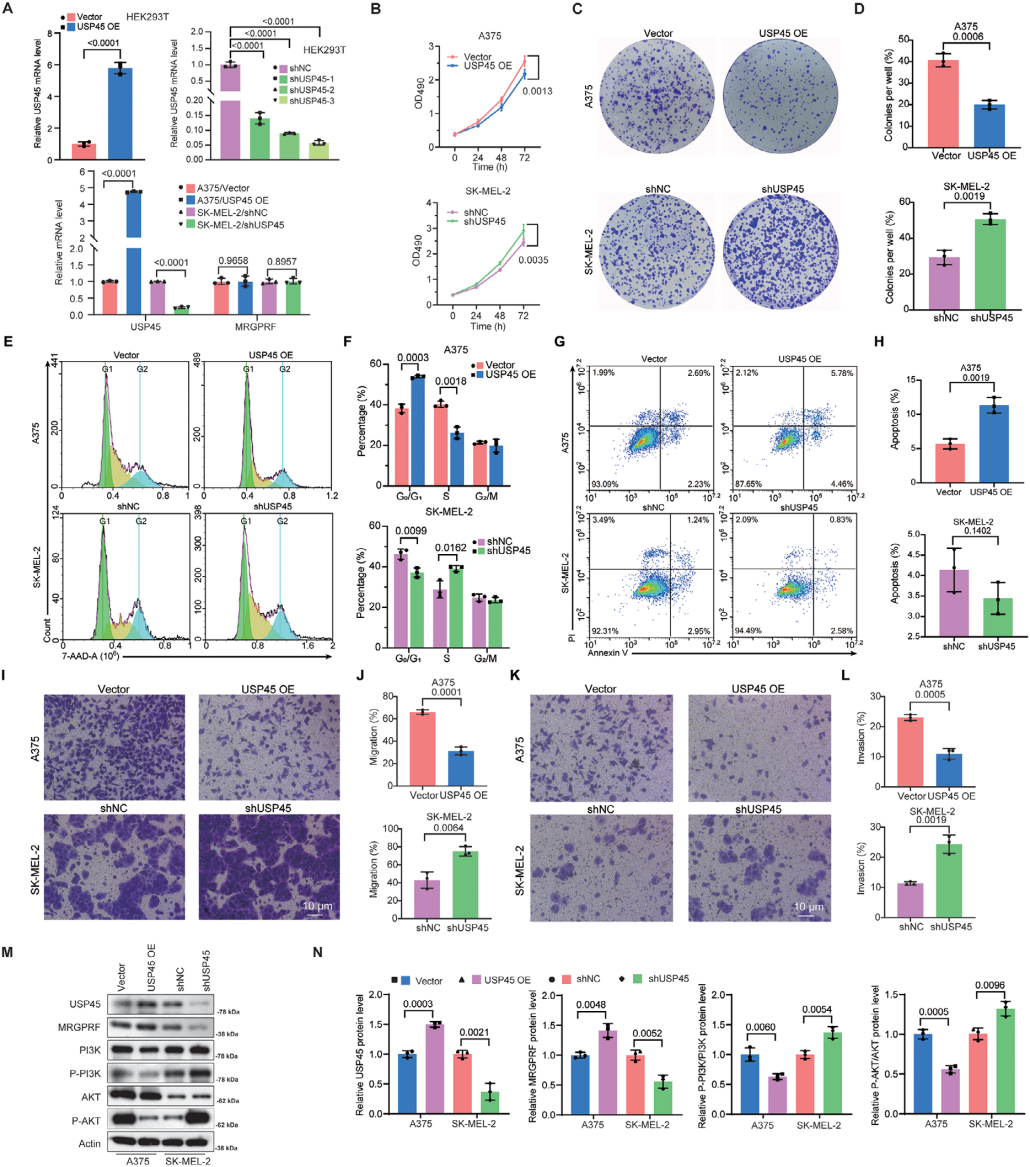
USP45 inhibits markers of melanoma malignancy in vitro and reduces the PI3K/AKT pathway. A) qPCR data showing the USP45 overexpression efficiency in HEK293T and A375 cells, along with USP45 knockdown efficiency in HEK293T and SK‐MEL‐2 cells. MRGPRF mRNA levels in melanoma cells with the indicated treatments were also present. B) MTT assay depicting the influence of USP45 overexpression or silencing on the viability of melanoma cells. C) Colony formation assay outcomes illustrating the impact of USP45 overexpression or deficiency on the colony formation ability of A375 or SK‐MEL‐2 cells. D) Statistical analysis of clone formation assay in (C). E) 7‐AAD assay results indicating the effect of USP45 overexpression or depletion on the cell cycle of melanoma cells. F) Statistical analysis of the 7‐AAD assay results present in (E). G) Annexin V/PI staining data showing the influence of excessive USP45 or USP45 depletion on the apoptosis of cells. H) Statistical analysis of the data shown in (G). I,K) Transwell assay results depicting the effect of USP45 overexpression or knockdown on the migration (I) and invasion (K) of A375 or SK‐MEL‐2 cells. J,L) Quantification of the data shown in (I) and (K). M) Western blot data illustrating the levels of USP45, MRGPRF, PI3K, P‐PI3K, AKT, and P‐AKT in control, USP45‐overexpressing, or USP45‐deficient melanoma cells. N) Statistical bar graph of Western blot data in (M). Data from two groups were analyzed using the unpaired bilateral Student's *t*‐test, while data from three or more groups were analyzed using one‐way ANOVA followed by Tukey's HSD.

To assess the function of USP45 in melanoma cells, we overexpressed USP45 in A375 cells and knocked down USP45 in SK‐MEL‐2 cells, as A375 and SK‐MEL‐2 cells express the lowest and highest levels of USP45, respectively, among the melanoma cells we examined. qPCR results showed that USP45 can be successfully overexpressed or knocked down in HEK293T cells (**Figure** **3**A). Consistent with this, qPCR and Western blot data confirmed that USP45 can be overexpressed in A375 cells and efficiently depleted in SK‐MEL‐2 cells (Figure 3A,M,N)). MTT assay data revealed that overexpression of USP45 impaired the viability of A375 cells, while USP45 deficiency exhibited the opposite effect on SK‐MEL‐2 cells (Figure 3B). In line with this, the findings of the colony formation experiment showed that an excessive amount of USP45 increased the ability of A375 cells to form colonies, while reducing the expression of USP45 disrupted the colony formation of SK‐MEL‐2 cells (Figure 3C,D). Moreover, overexpression of USP45 caused more A375 cells to be arrested in the G0/G1 stage, while USP45 deficiency facilitated the progression of SK‐MEL‐2 cells to the S stage (Figure 3E,F). Interestingly, we observed that USP45 overexpression promoted the apoptosis of A375 cells, whereas the reduction of USP45 had no significant impact on the apoptosis of SK‐MEL‐2 cells (Figure 3G,H). Furthermore, Transwell assay outcomes illustrated that the invasion and migration of A375 cells were strengthened by USP45 overexpression, whereas the invasion and migration of SK‐MEL‐2 cells were mitigated upon USP45 depletion (Figure 3I–L).

Then, we investigated whether USP45 has a similar effect on the PI3K/AKT pathway. Notably, USP45 overexpression or knockdown had minimal influence on the mRNA level of MRGPRF in A375 or SK‐MEL‐2 cells, respectively (Figure 3A); however, the MRGPRF protein level was elevated in USP45‐overexpressing A375 cells and decreased in USP45‐deficient SK‐MEL‐2 cells. The levels of P‐PI3K and P‐AKT were upregulated in USP45‐overexpressing A375 cells and reduced in USP45‐deficient SK‐MEL‐2 cells (Figure 3M,N). Collectively, these observations demonstrate that USP45 acts as a suppressor of melanoma, possibly by stabilizing MRGPRF.

### 3.4 The Catalytic Domain Is Required for USP45 to Fulfill Its Anticancer Function in Melanoma Cells

**Figure 4:**
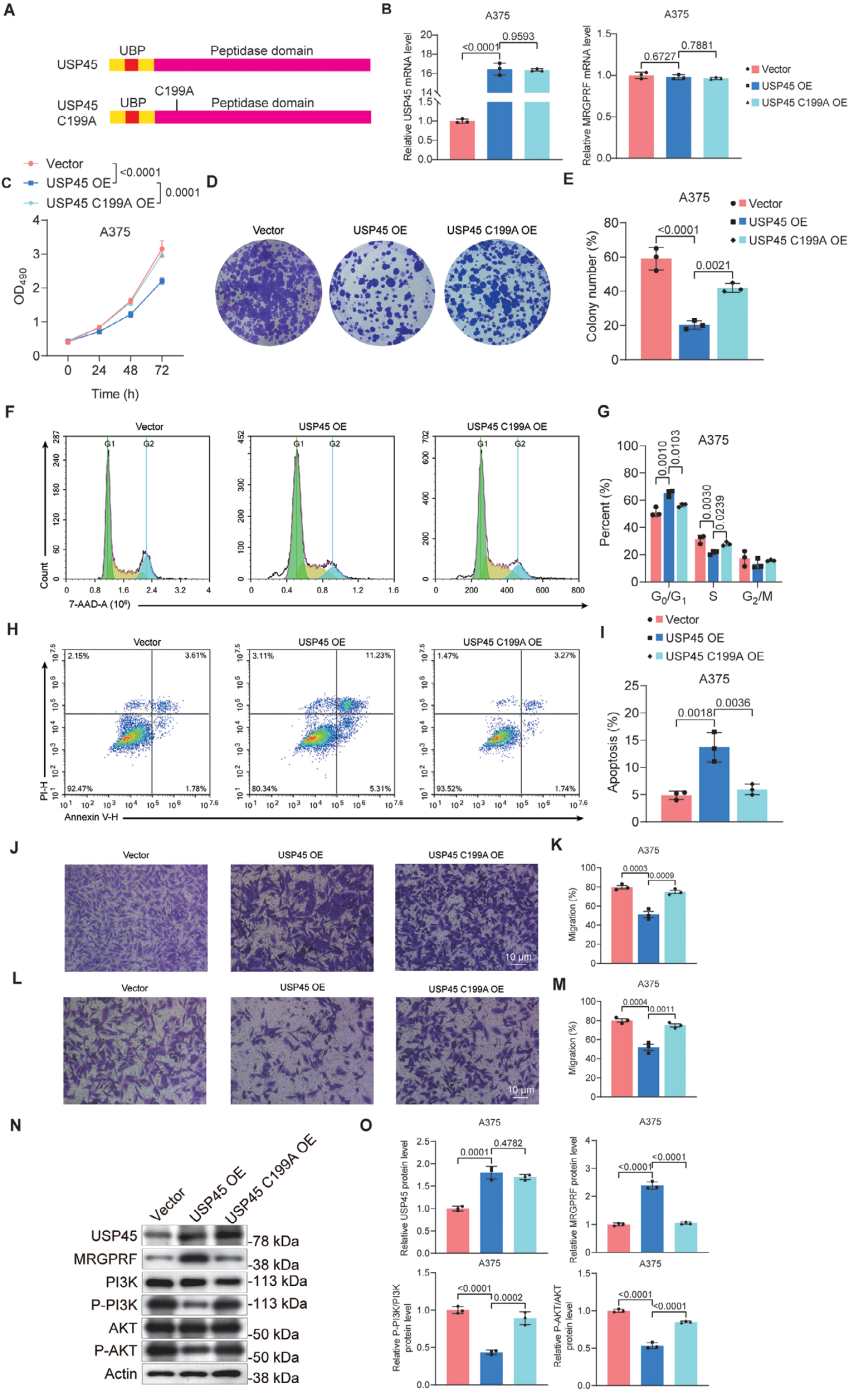
USP45's catalytic domain is vital for its antimelanoma function. A) Schemes showing the structure of wildtype USP45 and USP45 C199A. B) qPCR data comparing the impact of wildtype USP45 or USP45 C199A overexpression on MRGPRF mRNA level in A375 cells. C) MTT assay results demonstrating the viability of A375 cells overexpressing wildtype USP45 or USP45 C199A. D) Colony formation assay data depicting the colony formation ability of A375 cells overexpressing wildtype USP45 or USP45 C199A. E) Statistical analysis of clone formation assay in (D). F) 7‐AAD staining outcomes showing the cell cycle of A375 cells overexpressing wildtype USP45 or USP45 C199A. G) Quantification of the data present in (F). H) Annexin V/PI staining results depicting the apoptosis status of A375 cells overexpressing wildtype USP45 or USP45 C199A. I) Statistical analysis of the data shown in (H). J,L) Transwell assay data illustrating the migration (J) and invasion (L) of A375 cells overexpressing wildtype USP45 or USP45 C199A. K,M) Statistical analysis of the data shown in (J) and (L). N) Western blot data indicating the levels of USP45, MRGPRF, PI3K, P‐PI3K, AKT, and P‐AKT in A375 cells overexpressing wildtype USP45 or USP45 C199A. O) Quantification of data shown in (N). *p* values were determined by unpaired bilateral Student's *t*‐tests.

Because USP45 is a DUB that removes the ubiquitin chain from its substrates, we tested whether its catalytic domain is required for its anticancer function in melanoma cells. To block USP45's catalytic function, we generated a mutant USP45, USP45 C199A,^[^ ^39^ ^]^ in which the cysteine (C) at position 199 was mutated to alanine (A) (**Figure** **4**A). qPCR results indicated that the C199A mutation did not affect the mRNA or protein levels of USP45 in A375 cells. While USP45 C199A did not influence MRGPRF mRNA expression, it was less efficient in stabilizing the MRGPRF protein compared to wildtype USP45, as shown by Western blot results (Figure 4B,N,O). Next, we compared the impact of wildtype USP45 and USP45 C199A on the viability, colony formation ability, cell cycle, apoptosis, invasion, and migration of A375 cells. The results revealed that the antimelanoma function of USP45 was substantially impaired by the C199A mutation (Figure 4C‐M). Additionally, USP45 C199A‐overexpressing A375 cells exhibited higher PI3K/AKT pathway activity, as demonstrated by Western blot data (Figure 4N,O). Together, these findings support the notion that the catalytic function of USP45 is necessary for its antimelanoma activity.

### 3.5 The Catalytic Domain of USP45 Interacts with MRGPRF's N‐Terminal

**Figure 5:**
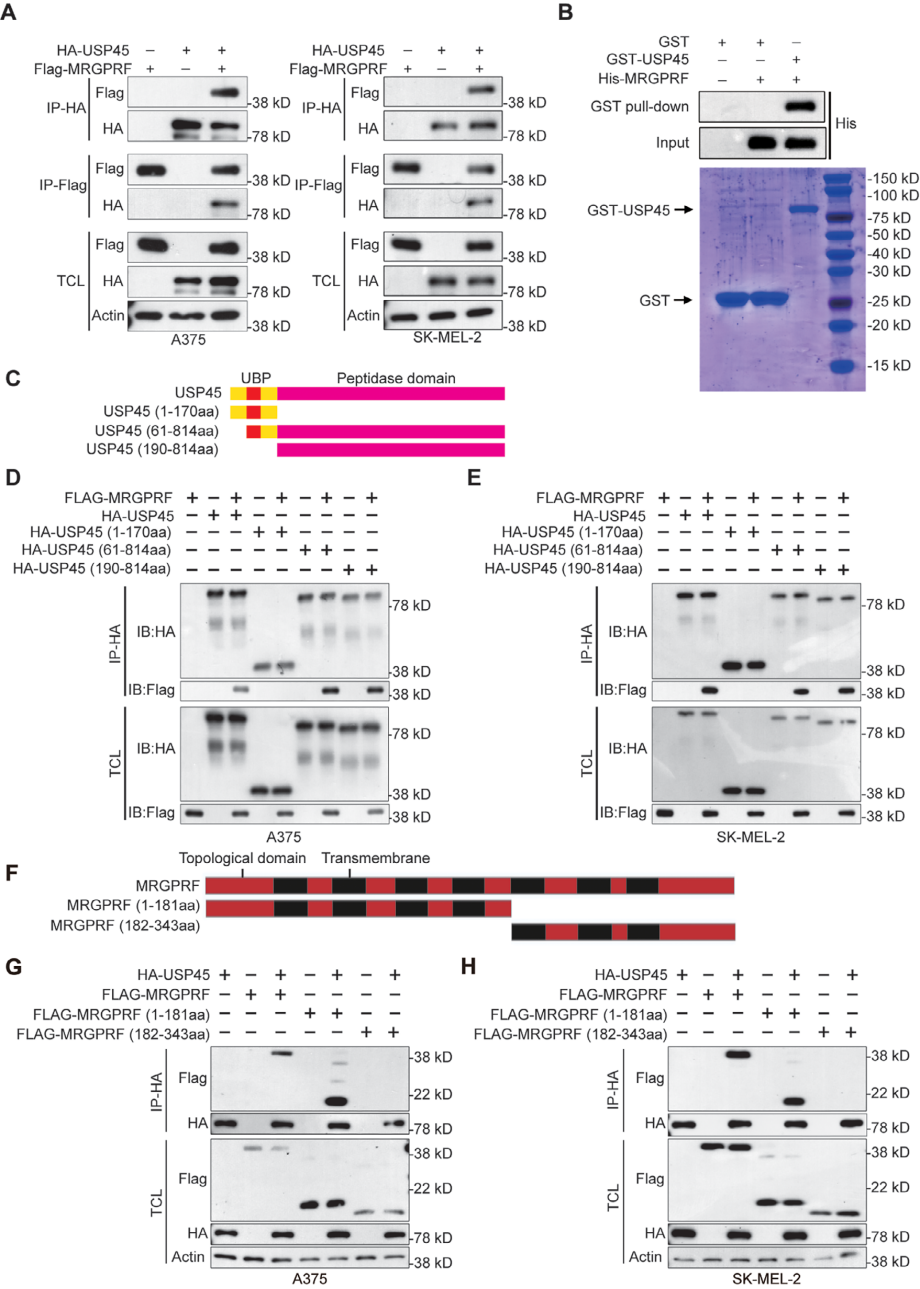
USP45 binds to MRGPRF's N‐terminal by its catalytic domain. A) Co‐IP data showing the interaction between USP45 and MRGPRF in A375 and SK‐MEL‐2 cells. B) GST pull‐down assay outcome indicating the direct binding of USP45 to MRGPRF. C) Schemes depicting the structure of the indicated USP45 isoforms. D,E) Co‐IP results illustrating the interaction between MRGPRF and the indicated USP45 isoforms in A375 and SK‐MEL‐2 cells. F) Schemes showing the structure of MRGPRF isoforms. G,H) Co‐IP data demonstrating the interaction between USP45 and the indicated MRGPRF isoforms in A375 and SK‐MEL‐2 cells. TCL represents total cell lysate.

Since USP45 stabilizes MRGPRF, we sought to determine whether USP45 interacts with MRGPRF. Co‐IP assay and SmBiT–LgBiT PPI luciferase assay results indicate that USP45 and MRGPRF can interact with each other in A375 and SK‐MEL‐2 cells (**Figures** **5**A and S2 (Supporting Information)). Supporting this finding, GST pull‐down assay outcomes showed that USP45 directly binds to MRGPRF (Figure 5B). To identify which domain of USP45 binds to MRGPRF, we generated the following USP45 truncation mutants: USP45 (1‐170aa) lacking the peptidase domain, USP45 (61‐814aa) having a truncated UBP domain, and USP45 (190‐814aa) lacking the entire UBP domain (Figure 5C). Western blot data confirmed that all these truncated USP45 mutants can be expressed by A375 and SK‐MEL‐2 cells. However, only USP45 (61‐814aa) and USP45 (190‐814aa), but not USP45 (1‐170aa), can interact with MRGPRF, as evidenced by co‐IP assay results (Figure 5D,E). Next, we produced two MRGPRF truncates: MRGPRF (1‐181aa), which lacks the C‐terminal, and MRGPRF (182‐343aa), which lacks the N‐terminal (Figure 5F). Co‐IP assay data revealed that MRGPRF (1‐181aa), but not MRGPRF (182‐343aa), interacts with USP45 (Figure 5G,H). These results illustrate that the catalytic domain is required for USP45 to interact with the N‐terminal of MRGPRF.

### 3.6 USP45 Stabilizes MRGPRF and Decreases Its K63‐Linked Ubiquitination in Melanoma Cells

**Figure 6:**
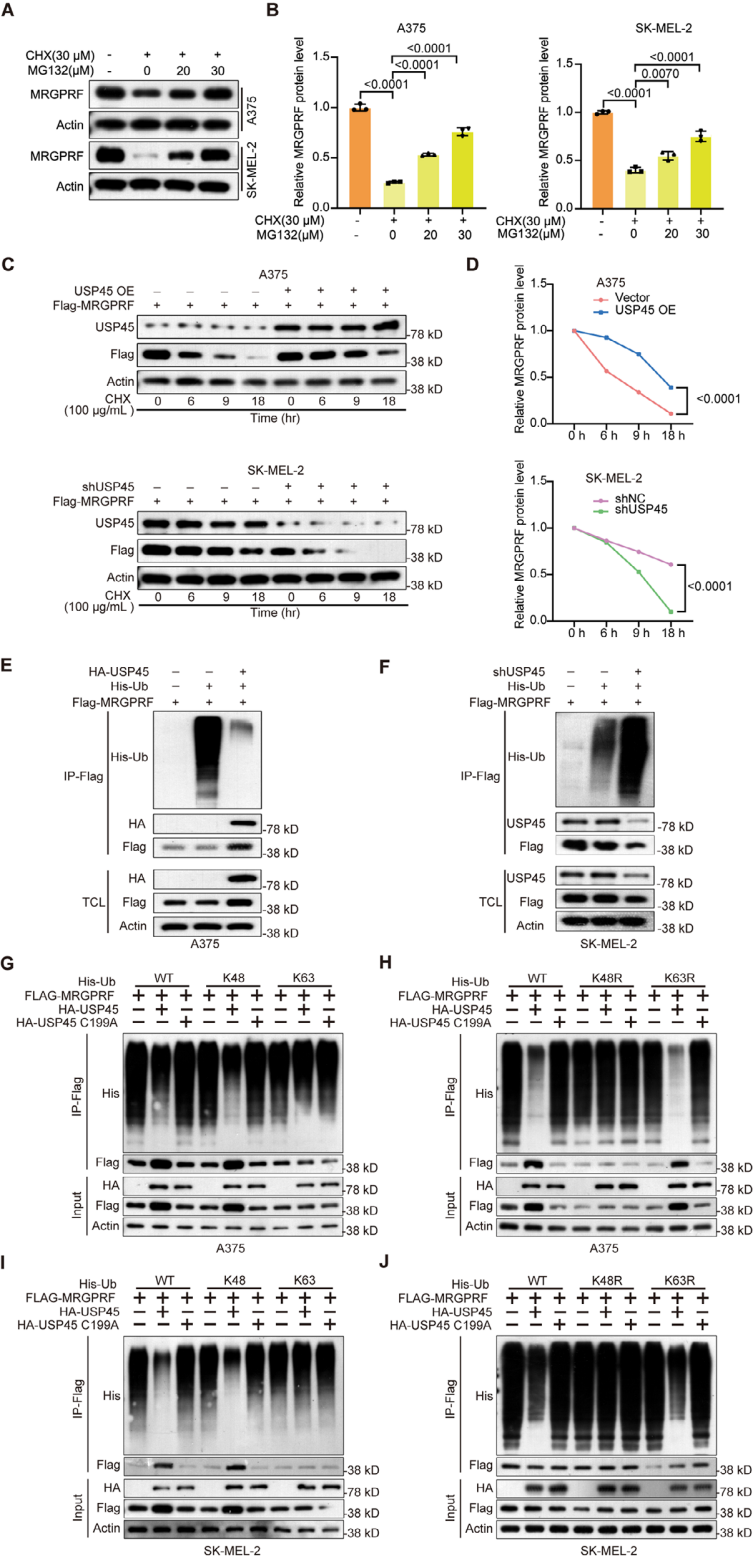
USP45 stabilizes MRGPRF by removing its K63‐linked ubiquitination in melanoma cells. A) Western blot data showing levels of MRGPRF in control A375 and SK‐MEL‐2 cells and their counterparts treated with CHX and MG132. B) Quantification of the Western blot data shown in (A). One‐way ANOVA followed by Tukey's HSD was utilized for *p* value calculation. C) Western blot data depicting the impact of USP45 overexpression or knockdown on the degradation of MRGPRF in A375 or SK‐MEL‐2 cells in the presence of CHX. D) Statistical analysis of the Western blot present in (C). Two‐way ANOVA was used to evaluate the statistical significance. E,F) IP assay outcomes illustrating the effect of USP45 overexpression or knockdown on the ubiquitination of MRGPRF in A375 or SK‐MEL‐2 cells. G,H) IP assay data showing the ubiquitination of MRGPRF in A375 cells with the indicated treatments in the presence of His‐Ub K63 or His‐Ub K48 (G), or His‐Ub K63R or His‐Ub K48R (H). I,J) IP assay data indicating the ubiquitination of MRGPRF in SK‐MEL‐2 cells with the indicated treatments in the presence of His‐Ub K63 or His‐Ub K48 (I), or His‐Ub K63R or His‐Ub K48R (J). TCL, total cell lysate.

To delineate the mechanism by which USP45 stabilizes MRGPRF in melanoma cells, we treated A375 and SK‐MEL‐2 cells with protein synthesis inhibitor CHX and proteasome inhibitor MG132. These chemicals enabled us to investigate the impact of proteasomal degradation on the existing MRGPRF protein. Western blot data indicated that MG132 stabilizes MRGPRF (**Figure** **6**A,B), confirming the essential role of the ubiquitin–proteasome system in regulating the stability of MRGPRF. Next, we tested whether USP45 prevents the degradation of MRGPRF in CHX‐treated cells. Western blot data uncovered that excessive USP45 reduced the degradation of MRGPRF protein in A375 cells, while depletion of USP45 accelerated MRGPRF degradation in SK‐MEL‐2 cells (Figure 6C,D).

Since USP45 is a DUB that stabilizes MRGPRF, we investigated the impact of USP45 on the ubiquitination level of MRGPRF. Western blot results showed that overexpression of USP45 significantly decreased the ubiquitination of MRGPRF in A375 cells, while USP45 depletion led to a remarkable increase in MRGPRF ubiquitination in SK‐MEL‐2 cells (Figure 6E,F). Considering that ubiquitination can be linked to multiple lysine residues in ubiquitin, we evaluated the effect of wildtype USP45 and USP45 C199A on K63‐ or K48‐linked ubiquitination, the two most common forms of ubiquitination, in MRGPRF protein. We generated His‐Ub K63, His‐Ub K63R, His‐Ub K48, and His‐Ub K48R constructs. In His‐Ub K63 or K48, only the K63 or K48 residue was retained while the other lysine residues of ubiquitin were mutated to arginine (R) residues. By contrast, in His‐Ub K63R or K48R, only the K63 residue or K48 residue was mutated to arginine, while the remaining lysine residues of ubiquitin were not altered. By performing co‐IP assays, we observed that mutation of K63 to arginine in ubiquitin abolished USP45‐mediated deubiquitination of MRGPRF in both cells, while the K48R mutation of ubiquitin had minimal influence on USP45's ability to deubiquitinate MRGPRF (Figure 6G–J). Notably, USP45 C199A displayed no obvious effect on the ubiquitination and degradation of MRGPRF, further indicating the pivotal role of USP45's catalytic domain in deubiquitinating MRGPRF (Figure 6G–J and Figure S3A–D (Supporting Information)). Collectively, these findings demonstrate that USP45 enhances the stability of MRGPRF in melanoma cells, likely by eliminating its K63‐linked ubiquitination.

### 3.7 MRGPRF Is Vital for USP45's Anticancer Function in Melanoma Cells

**Figure 7:**
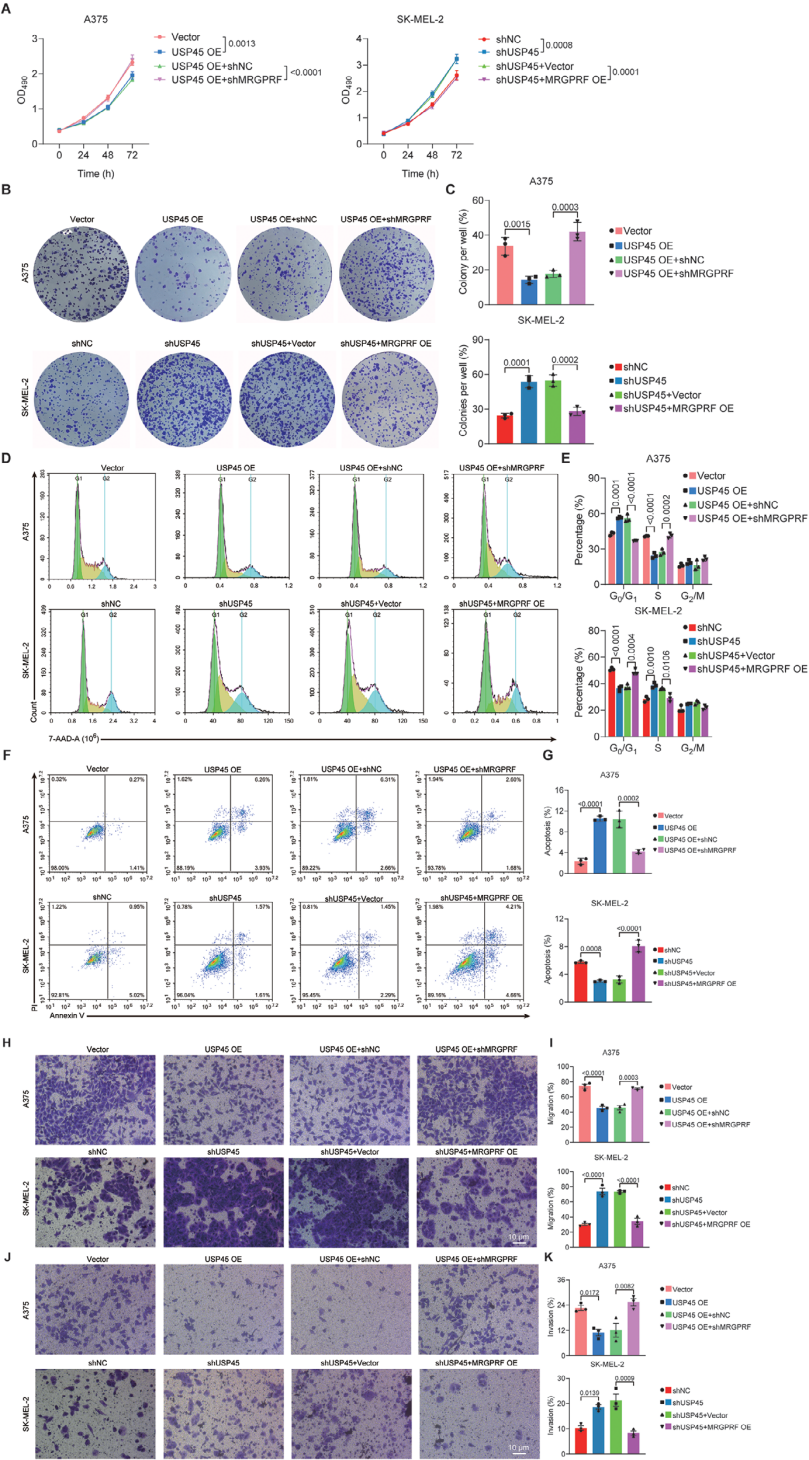
MRGPRF mediates the anticancer function of USP45 in melanoma cells. A) MTT assay results showing the influence of MRGPRF depletion on the viability of USP45‐overexpressing A375 cells and the impact of MRGPRF overexpression on the viability of USP45‐deficient SK‐MEL‐2 cells. B) Colony formation assay data depicting the effect of MRGPRF knockdown on the colony‐forming ability of USP45‐overexpressing A375 cells and the impact of MRGPRF overexpression on the colony‐forming ability of USP45‐deficient SK‐MEL‐2 cells. C) Quantification of the data present in (B). D) 7‐AAD assay outcomes indicating the influence of MRGPRF silencing on the cell cycle of USP45‐overexpressing A375 cells and the impact of overdosed MRGPRF on the cell cycle of USP45‐deficient SK‐MEL‐2 cells. E) Quantification of the data shown in (D). F) Annexin V/PI staining results demonstrating the impact of MRGPRF knockdown on the apoptosis of USP45‐overexpressing A375 cells and the impact of MRGPRF overexpression on the apoptosis of USP45‐deficient SK‐MEL‐2 cells. G) Quantification of the data present in (F). H,J) Transwell assay outcomes showing the effect of MRGPRF deficiency on the migration and invasion of USP45‐overexpressing A375 cells and the impact of excessive MRGPRF on the migration and invasion of USP45‐deficient SK‐MEL‐2 cells. I,K) Statistical analysis of the data shown in (H) and (J). One‐way ANOVA followed by Tukey's HSD was utilized for *p* value calculation.

Given that both USP45 and MRGPRF repress melanoma and that USP45 stabilizes MRGPRF, we investigated whether MRGPRF acts downstream of USP45 in inhibiting melanoma. To test this hypothesis, we depleted MRGPRF in USP45‐overexpressing A375 cells and overexpressed MRGPRF in USP45‐deficient SK‐MEL‐2 cells. We then assessed the malignant behaviors of these cells and their control counterparts. The efficiency of shMRGPRFs was initially validated in HEK293T cells (Figure S4, Supporting Information). MTT assay data revealed that MRGPRF depletion abolished the antiviability function of excessive USP45 in A375 cells, while MRGPRF overexpression reversed the increased viability resulting from USP45 deficiency (**Figure** **7**A). Consistently, colony formation experiments consistently demonstrated that reducing the expression of MRGPRF significantly promoted the colony formation ability of USP45‐overexpressing A375 cells, while MRGPRF overexpression substantially reduced the colony formation ability of USP45‐deficient SK‐MEL‐2 cells (Figure 7B,C). Moreover, the cell cycle progression of USP45‐overexpressing A375 cells and USP45‐deficient SK‐MEL‐2 cells was promoted and impeded by MRGPRF knockdown and overexpression, respectively, as evidenced by 7‐AAD staining results (Figure 7D,E). Notably, knockdown of MRGPRF impaired the antiapoptotic effect of USP45 in A375 cells, whereas excessive MRGPRF elevated the apoptosis of USP45‐deficient SK‐MEL‐2 cells (Figure 7F,G). Additionally, the invasion and migration of USP45‐overexpressing A375 cells and USP45‐deficient SK‐MEL‐2 cells were, respectively, enhanced and attenuated by MRGPRF depletion and overexpression (Figure 7H–K). These results provide compelling evidence that MRGPRF is essential in mediating USP45's antimelanoma effect in vitro.

### 3.8 MRGPRF Depletion Reverses the Inhibitory Effect of USP45 on the PI3K/AKT Signaling Pathway in Melanoma Cells

**Figure 8:**
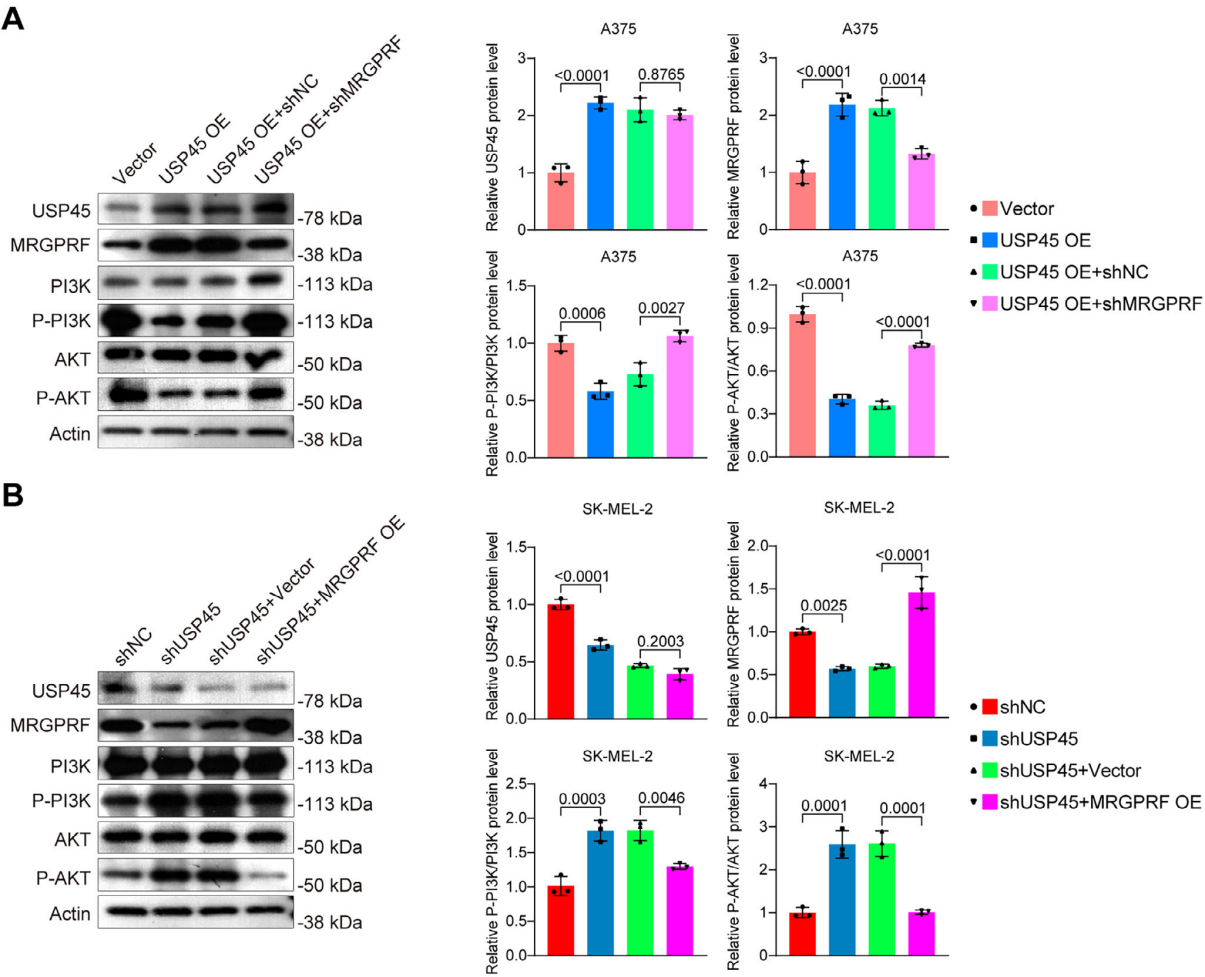
MRGPRF depletion mitigates the inhibitory effect of USP45 on the PI3K/AKT signaling pathway. A,B) Western blot data illustrating the levels of USP45, MRGPRF, PI3K, P‐PI3K, AKT, and P‐AKT in A375 cells (A) and SK‐MEL‐2 cells (B) with the indicated treatments. One‐way ANOVA followed by Tukey's HSD was utilized for *p* value calculation.

To determine whether MRGPRF acts downstream of USP45 in inhibiting PI3K/AKT pathway activation, we silenced MRGPRF in USP45‐overexpressing A375 cells and overexpressed MRGPRF in USP45‐deficient SK‐MEL‐2 cells. Western blot results showed that MRGPRF deficiency caused a significant increase in P‐AKT and P‐PI3K levels in USP45‐overexpressing A375 cells (**Figure** **8**A). By contrast, MRGPRF overexpression exhibited an opposite effect on the P‐AKT and P‐PI3K levels in USP45‐deficient SK‐MEL‐2 cells (Figure 8B). These results suggest that MRGPRF plays a crucial role in mediating USP45's inhibition of the PI3K/AKT pathway.

### 3.9 USP45 Overexpression Inhibits the Growth of Melanoma Xenograft

**Figure 9:**
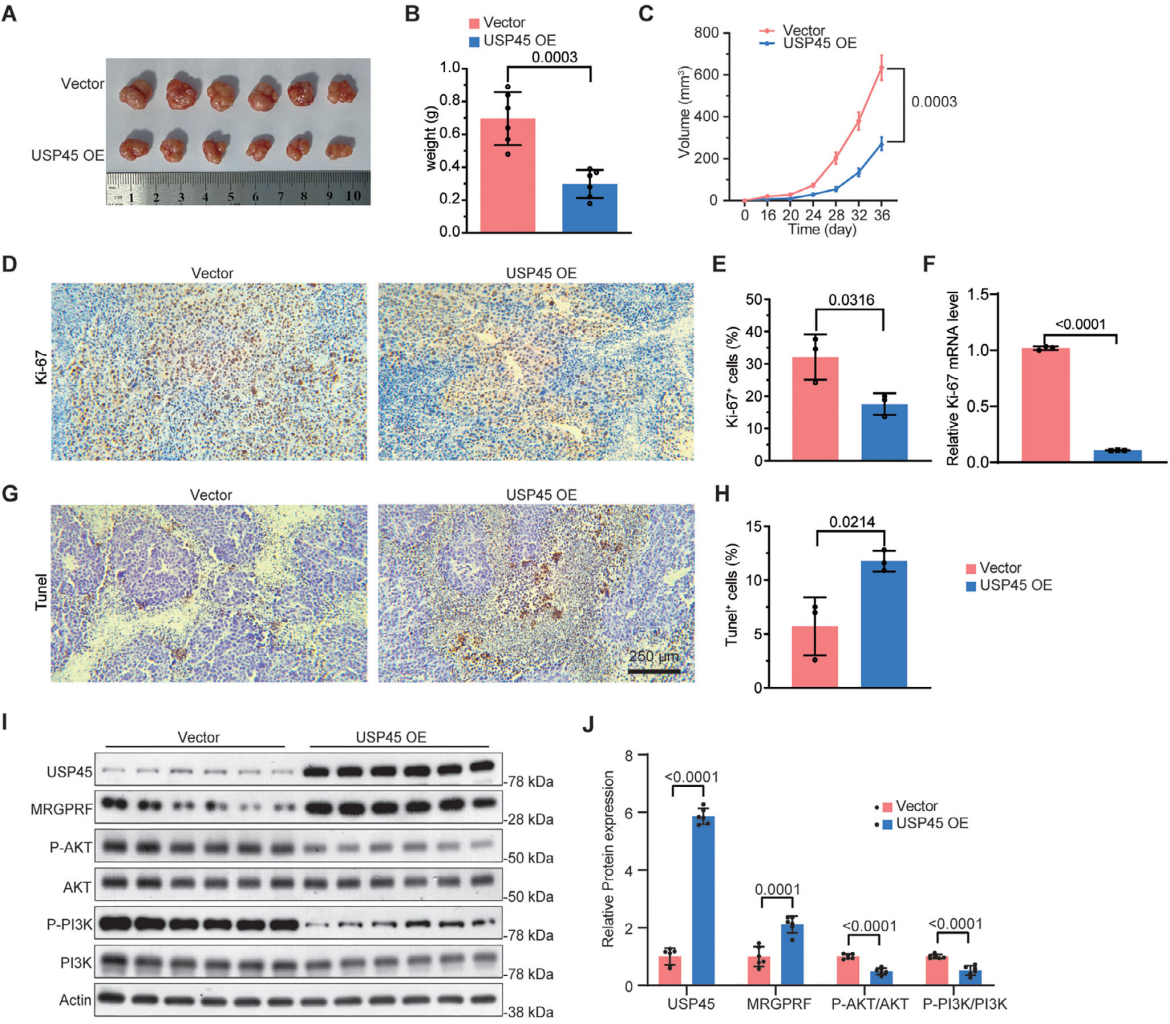
Excessive USP45 represses melanoma growth and induces apoptosis in vivo. A) Whole‐mount view of xenograft tumors developed from control or USP45‐overexpressing A375 stable cells. B,C) Quantification of the weight (B) and volume (C) of control or USP45‐overexpressing tumors (*n* = 6). D) IHC data showing the cell proliferation within control or USP45‐overexpressing tumors. E) Statistical analysis of the data shown in (D) (*n* = 3). F) qPCR results indicating the mRNA expression of human Ki‐67 in control or USP45‐overexpressing tumors (*n* = 3). G) TUNEL assay results depicting the apoptosis within control or USP45‐overexpressing tumors. H) Statistical analysis of the TUNEL assay data shown in (F) (*n* = 3). I) Western blot outcomes illustrating the expression of USP45, MRGPRF, PI3K, P‐PI3K, AKT, and P‐AKT in control or USP45‐overexpressing tumors. J) Quantification of data present in (H) (*n* = 6). *p* values were determined by unpaired bilateral Student's *t*‐tests.

To assess whether USP45 inhibits melanoma progression in vivo, we constructed USP45‐overexpressing stable A375 cells and injected them or control A375 cells into nude mice to establish xenograft models. The xenograft tumors formed from USP45‐overexpressing cells exhibited a notable reduction in size compared to those originating from control A375 cells (**Figure** **9**A–C). In support of this finding, IHC analysis of Ki‐67 protein and qPCR for human‐specific Ki‐67 mRNA, a proliferation marker, showed that USP45‐overexpressing xenograft tumors were less proliferative than control tumors (Figure 9D–F). Moreover, USP45‐overexpressing tumors exhibited more apoptosis than control tumors, as evidenced by TUNEL assay results (Figure 9G,H). Additionally, Western blot outcomes confirmed the overexpression of USP45 and elevated MRGPRF levels in USP45‐overexpressing xenograft tumors, which also exhibited reduced activity of the PI3K/AKT pathway compared to control tumors (Figure 9I–J). These results suggest that USP45 acts as a suppressor of melanoma in vivo, likely through the stabilization of MRGPRF and subsequent inhibition of the PI3K/AKT pathway.

### 3.10 USP45 Negatively Regulates the Expression of MYC and Snail in Melanoma Cells

To investigate whether the antimelanoma effect of USP45 is mediated by additional substrates, we examined its influence on the expression of MYC and Snail, which are known substrates of USP45 and are stabilized by it in other cancers.^[^ ^40^, ^41^ ^]^ Western blot analysis revealed that MYC and Snail expressions were suppressed by USP45 overexpression in A375 cells, whereas their levels were elevated in USP45‐deficient SK‐MEL‐2 cells (Figure S5A, Supporting Information). Consistently, MYC and Snail protein levels were also reduced in USP45‐deficient xenograft tumors compared to their controls, as demonstrated by Western blot data (Figure S5B, Supporting Information). These findings indicate that, unlike in other cancers, USP45 does not stabilize MYC and Snail in melanoma cells but instead inhibits their expression.

## 4 Discussion

The low expression of MRGPRF in melanoma tissues may result from hypermethylation of its promoter.^[^ ^19^ ^]^ However, its expression level may also be controlled at the posttranslational level. Supporting this theory, we observed that USP45 affects the stability of MRGPRF protein, indicating that a dysregulated ubiquitin–proteasome system may also contribute to reduced MRGPRF expression and melanoma tumorigenesis.

Several USPs, such as USP4 and USP7, have been implicated in promoting melanoma development, highlighting the essential roles of ubiquitination regulators in this disease. Our finding that USP45 functions as a melanoma suppressor contrasts with these oncogenic USPs, suggesting that the specific substrates targeted by USPs determine their roles in melanoma. For example, USP45 has been shown to promote stemness and drug resistance in cervical cancer cells by stabilizing the oncogenic MYC.^[^ ^41^ ^]^ Similarly, USP45 functions as an oncogene in ovarian cancer cells, potentially through the stabilization of Snail.^[^ ^40^ ^]^ By contrast, our study demonstrates that USP45 stabilizes MRGPRF, which acts as a melanoma suppressor. Additionally, USP45 and MRGPRF negatively regulate MYC and Snail expression in melanoma cells, possibly because they are melanoma suppressors, while MYC and Snail serve as oncogenes in melanoma.^[^ ^42^, ^43^ ^]^ Alternatively, the regulation of MYC and Snail by USP45 may involve other factors that are absent in melanoma cells. This indicates that the impact of USPs is highly context‐dependent, influenced by their interaction with specific substrates.

Additionally, our finding that USP45 mRNA and protein levels are downregulated in melanoma compared to noncancerous epidermis indicates that USP45 expression may be tightly controlled at multiple levels, such as transcriptional, translational, or posttranslational. It is possible that the promoter of USP45, like that of MRGPRF, is hypermethylated, leading to decreased expression in melanoma. Alternatively, USP45 mRNA could be targeted by an overexpressed miRNA, or the degradation of USP45 protein may be accelerated by abnormal ubiquitination. Further investigations are warranted to explore these possibilities.

Notably, although both USP45 and MRGPRF play critical roles in controlling melanoma development, their functions in melanocytes, the cells from which melanoma originates, remain undetermined. Additionally, it is unknown whether their expression levels in melanocytes differ from those in melanoma cells. The MRGPR receptors are known for their function in peripheral sensory neurons, which arise from neural crest cells, the same origin as melanocytes. Therefore, it is plausible that both MRGPRF and USP45 have roles in melanocytes as well. Comparing their expression levels and deciphering their functions in melanocytes would enhance our understanding of the USP45–MRGPRF axis in melanoma tumorigenesis. This comparison could shed light on the initial steps of melanoma development.

The catalytic domain of USP45 is critical for its function as a DUB, as the USP45 C199A mutant loses the ability to stabilize MRGPRF and suppress melanoma. Moreover, this domain appears to be required for binding to MRGPRF. Curiously, in different situations, USP45 forms a connection with ERCC1 through its brief acidic motif that is positioned outside of the catalytic domain.^[^ ^44^ ^]^ Similarly, the binding of USP45 to MYC in cervical cancer cells is also independent of its catalytic domain.^[^ ^41^ ^]^ The specific domain responsible for the USP45–Snail interaction remains unknown. MRGPRF is a multitransmembrane protein, and our data show that its N‐terminal binds to USP45. The extracellular N‐terminal of MRGRP receptors is responsible for agonist/ligand binding and is activated by proteases,^[^ ^45^, ^46^ ^]^ while their intracellular C‐terminal initiates downstream signal transduction.^[^ ^47^ ^]^ However, the precise domain and residues mediating the USP45–MRGPRF binding require further characterization. This deeper understanding could provide valuable insights into the molecular mechanisms by which USP45 influences its substrates and modulates melanoma progression.

While our study focuses on USPs that deubiquitinate MRGPRF, the E3 ligase responsible for ubiquitinating MRGPRF has not been identified. MRGPRF may exhibit multiple types of ubiquitination. We explored its K48‐ and K63‐linked ubiquitination by IP assays and discovered that USP45 likely stabilizes MRGPRF by removing its K63‐linked but not K48‐linked ubiquitination. However, this conclusion should be further validated through site‐specific ubiquitination analysis using mass spectrometry. Moreover, it remains possible that other DUBs may affect MRGPRF stability by removing K48‐linked ubiquitination. Additionally, there are other types of ubiquitination at additional lysine residues, such as K6, K11, and K27.^[^ ^32^ ^]^ Whether these noncanonical types of ubiquitination exist in MRGPRF and regulate its function, distribution, and stability in melanoma cells needs to be determined. Additional investigation is necessary to identify the E3 ligase and explore the potential roles of various types of ubiquitination in the regulation of MRGPRF. This could offer a more extensive comprehension of the molecular pathways that regulate the stability and activity of MRGPRF in melanoma cells.

Despite a lower expression of USP45 in melanoma cells compared to keratinocytes, our data reveal variation in USP45 expression across different melanoma cell lines. For instance, USP45 expression in SK‐MEL‐2 cells is substantially higher than in A375 cells. The mechanism underlying this discrepancy is unknown, but these cells seem to show different responses to changes in USP45 levels. In particular, knockdown of USP45 in SK‐MEL‐2 cells has no significant influence, while overexpression of USP45 in A375 cells enhances their apoptosis. It is likely that a low level of USP45 is sufficient to balance the survival‐promoting mechanisms in SK‐MEL‐2 cells.

It is noteworthy that the impact of overdosed USP45 on melanoma cells in vivo appears slightly milder than its effect observed in vitro. This discrepancy could be attributed to the complex tumor microenvironment, which may partially compromise the antimelanoma effects of USP45. Furthermore, our xenograft assay only demonstrates that excessive USP45 inhibits proliferation and induces apoptosis of melanoma in vivo. Whether the antimelanoma effect of USP45 is mediated by MRGPRF in these xenograft tumors, and whether USP45 overexpression represses melanoma metastasis, has not been tested. Additionally, the USP45–MRGPRF interaction in primary melanoma cells needs to be verified. Finally, the impact of USP45–MRGPRF axis in other signaling pathways that are involved in melanoma progression, such as the RAS/MAPK pathway, remains to be determined.

## 5 Conclusion

**Figure 10:**
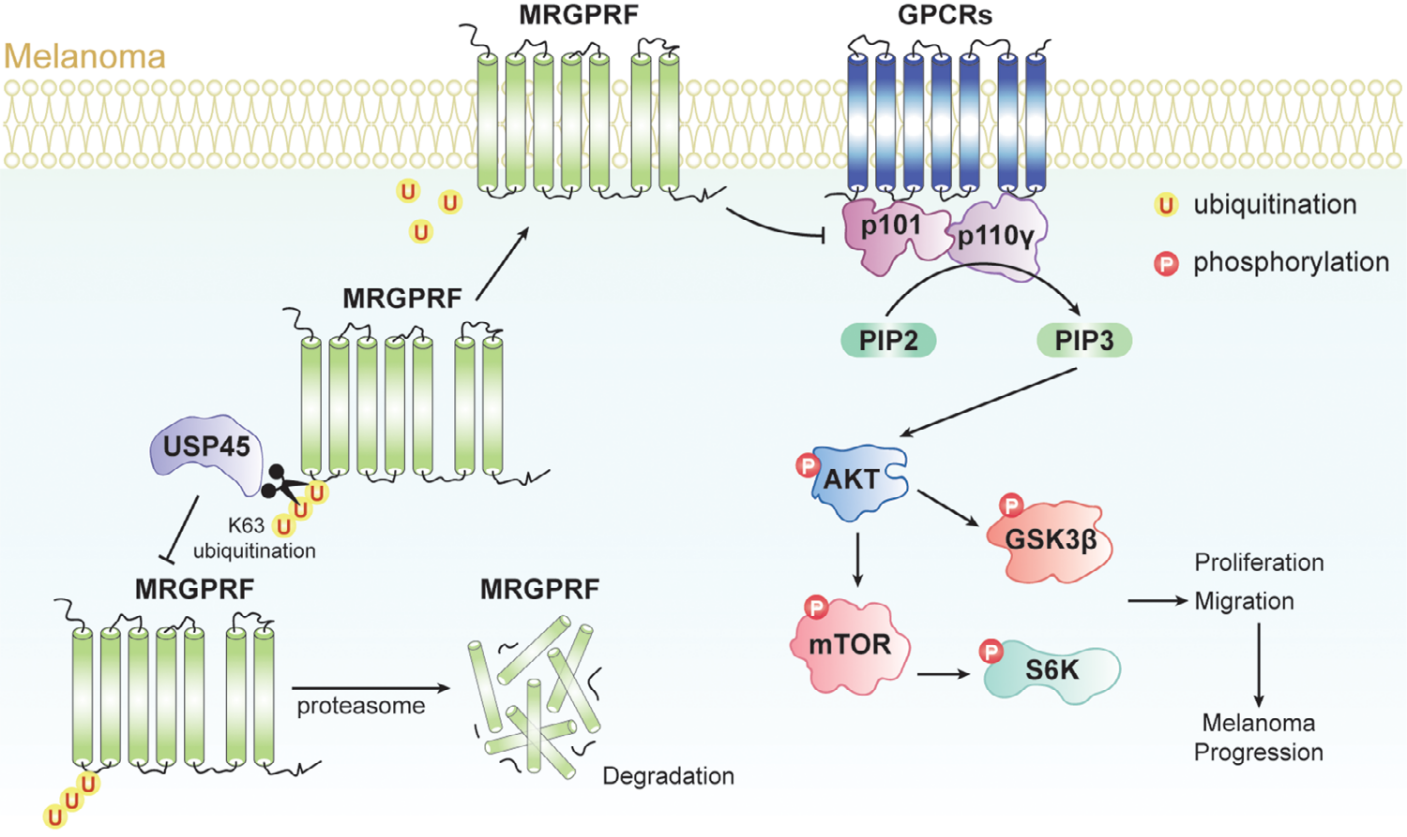
Schematic presentation of the role of USP45 in melanoma cells. MRGPRF is ubiquitinated by an uncharacterized E3 ligase, promoting its degradation via the proteasome. USP45 deubiquitinates and stabilizes MRGPRF, which in turn interferes with the formation of p110γ/p101 complex, thereby repressing the oncogenic PI3K/AKT pathway. This pathway inhibition leads to increased apoptosis and reduced proliferation, cell cycle progression, migration, and invasion of melanoma cells.

In summary, our data demonstrate that USP45 functions as a melanoma suppressor, at least in part, by stabilizing MRGPRF, thereby attenuating the PI3K/AKT pathway, inhibiting proliferation, inducing cell cycle arrest, promoting apoptosis, and mitigating the migration and invasion of melanoma cells (**Figure** **10**). These findings enhance our understanding of USP45's role in tumorigenesis and highlight the potential of targeting the USP45–MRGPRF axis as a strategy for melanoma treatment.

## Conflict of Interest

The authors declare no conflict of interest.

## Author Contributions

Conceptualization: W.C.Z., L.Y.C., J.Z., S.J.T.; data curation: W.C.Z., A.W.M.; formal Analysis: W.C.Z., L.Y.C., J.Z., A.W.M., S.J.T.; funding acquisition: W.C.Z.; investigation: W.C.Z., L.Y.C., J.Z.; methodology: W.C.Z., L.Y.C., J.Z., A.W.M., W.Q.S.; project administration: S.J.T.; resources: S.J.T.; supervision: S.J.T.; validation: L.Y.C., A.W.M., J.Z.; visualization: Y.W.Z., Z.X.T., J.R.G., Z.H.X., J.D.Z., S.J.T.; writing – original draft preparation: W.C.Z.; writing – review and editing: L.Y.C., J.Z., A.W.M., W.Q.S., Y.W.Z., Z.X.T., J.R.G., Z.H.X., J.D.Z., S.J.T.

## Supporting information



Supporting Information

Supplemental Figure 1

Supplemental Figure 2

Supplemental Figure 3

Supplemental Figure 4

Supplemental Figure 5

## Data Availability

The data that support the findings of this study are available from the corresponding author upon reasonable request.
