# Supplementary material for: USP45 Represses Melanoma Development by Deubiquitinating and Stabilizing Tumor Suppressor MRGPRF
Source: Adv Sci (Weinh). 2025 Aug 11;12(40):e03106. doi: 10.1002/advs.202503106 (PMC12561219; doi:10.1002/advs.202503106)
Supplement: Supplementary file 1 — Supporting Information [file ADVS-12-e03106-s003.docx]

**Supplementary information**

**Figure S1. MRGPRF expression is reduced in melanoma and positively correlates with USP45 expression.** (A) IHC data showing the MRGPRF expression in melanoma and control epidermis. (B) Quantification of the data shown in (A). P values were determined by unpaired bilateral Student’s t-test. (C) Correlation analysis results depicting the expression of MRGPRF and USP45 in melanoma tissues based on IHC results.

**Figure S2. USP45 directly interacts with MRGPRF in melanoma cells.** SmBiT-LgBiT PPI luciferase assay results showing the direct interaction between USP45 and MRGPRF in A375 and SK-MEL-2 cells.

**Figure S3. USP45 C199A is incapable of ubiquitinating or stabilizing MRGRPF.** (A) IP data showing the interaction between MRGPRF and wildtype USP45 or USP45 C199A in A375 cells. (B) IP results indicating the impact of wildtype USP45 or USP45 C199A on the ubiquitination of MRGPRF in A375 cells. (C) Western blot outcomes showing the influence of wildtype USP45 or USP45 C199A on the degradation of MRGPRF in A375 cells. (D) Quantification of the data shown in (C). TCL, total cell lysate. Two-way ANOVA followed by Tukey’s HSD was utilized for P value calculation.

**Figure S4. Lentiviral shMRGPRFs efficiently depleted MRGPRF in HEK293T cells**. qPCR data indicate that all three shRNAs targeting MRGPRF show high efficiency in HEK293T cells. One-way ANOVA followed by Tukey’s HSD was utilized for P value calculation.

**Figure S5. USP45 negatively regulates MYC and Snail expression in melanoma cells.** (A) Western blot data indicating the expression of MYC and Snail in A375 cells and SK-MEL-2 cells with the indicated treatments. (B) Western blot data showing the levels of MYC and Snail in control and USP45-overexpressing xenograft tumors.
