# Supplementary figures and images for: USP45 Represses Melanoma Development by Deubiquitinating and Stabilizing Tumor Suppressor MRGPRF
Source: Adv Sci (Weinh). 2025 Aug 11;12(40):e03106. doi: 10.1002/advs.202503106 (PMC12561219; doi:10.1002/advs.202503106)

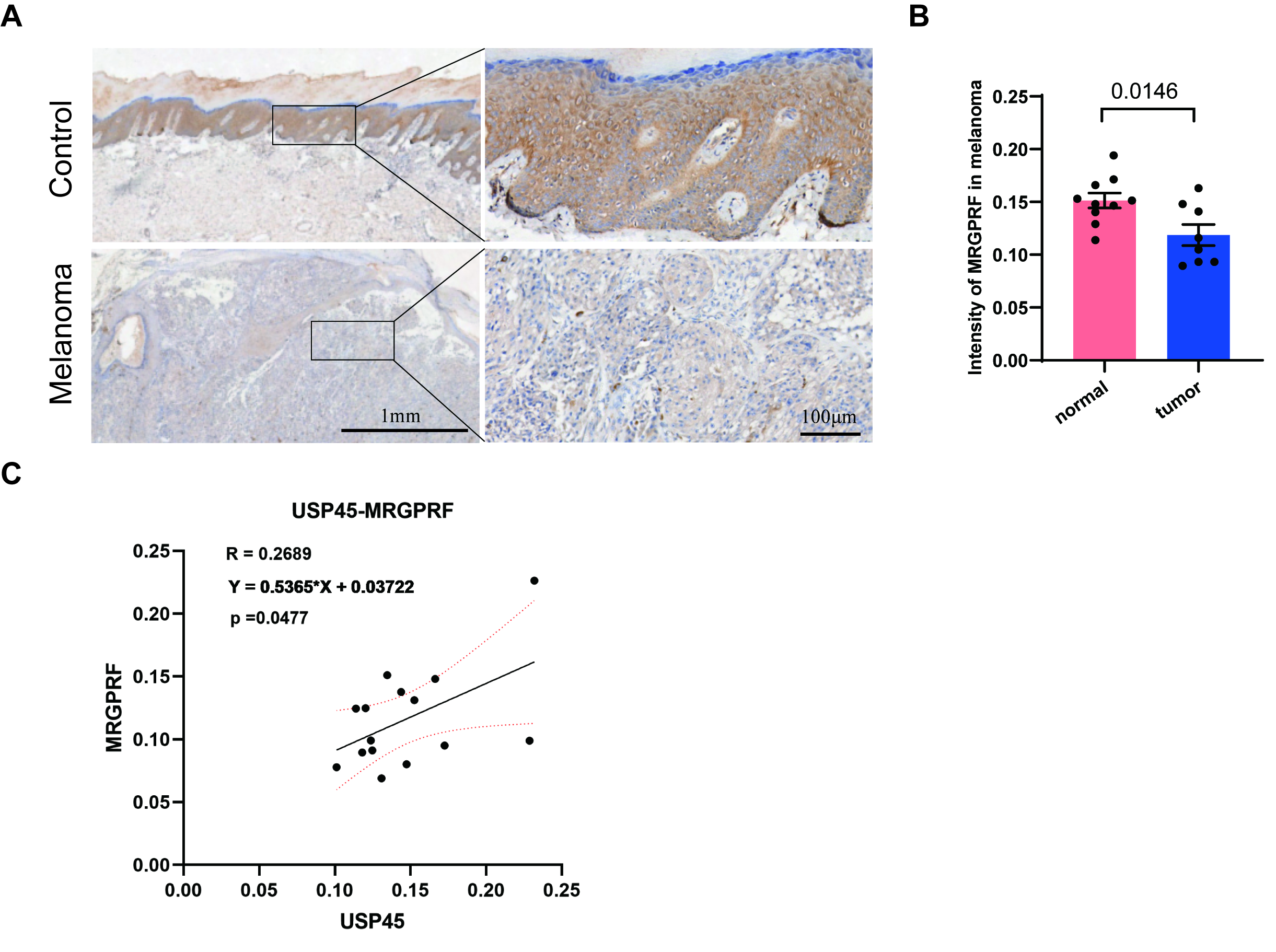

Supplement: Supplementary file 2 — Supplemental Figure 1 [file ADVS-12-e03106-s004.tif]

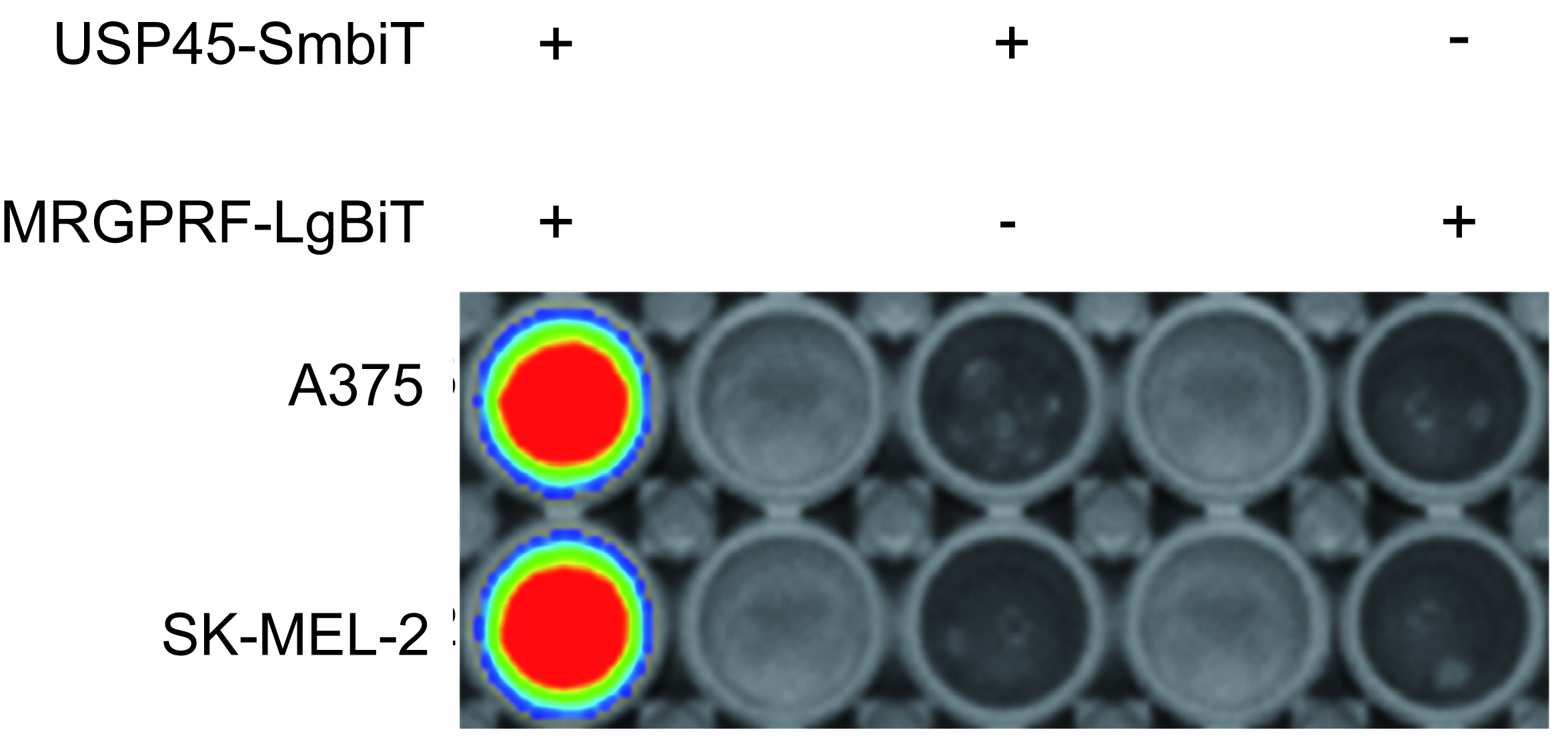

Supplement: Supplementary file 3 — Supplemental Figure 2 [file ADVS-12-e03106-s006.tif]

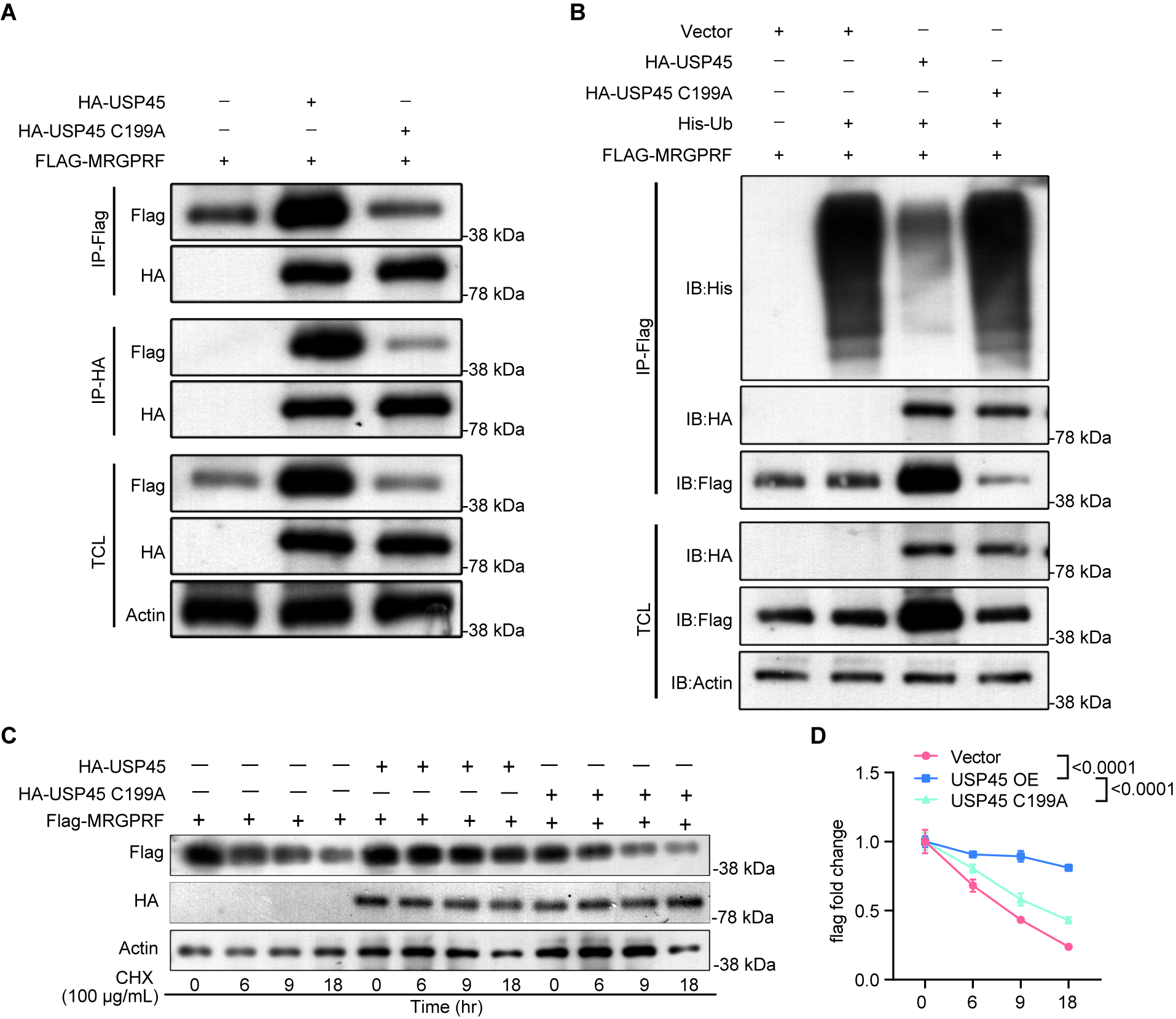

Supplement: Supplementary file 4 — Supplemental Figure 3 [file ADVS-12-e03106-s002.tif]

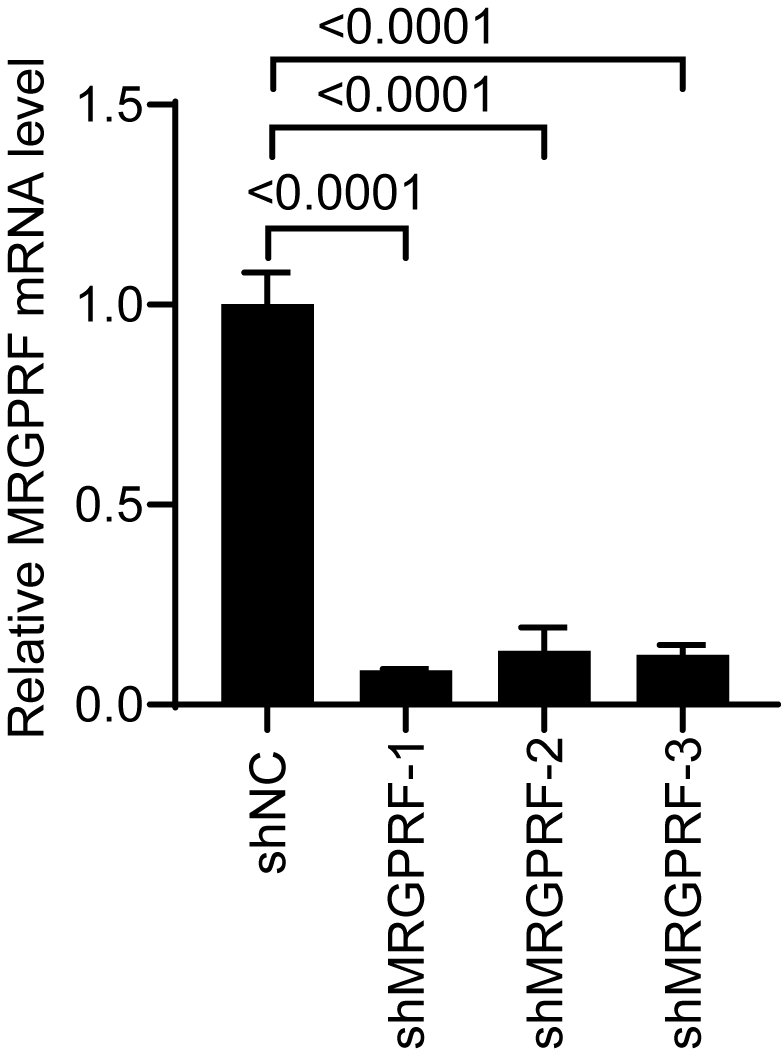

Supplement: Supplementary file 5 — Supplemental Figure 4 [file ADVS-12-e03106-s001.tif]
